# Microbial-Based Green Synthesis of Silver Nanoparticles: A Comparative Review of Bacteria- and Fungi-Mediated Approaches

**DOI:** 10.3390/ijms262010163

**Published:** 2025-10-19

**Authors:** Emir Akdaşçi, Furkan Eker, Hatice Duman, Mikhael Bechelany, Sercan Karav

**Affiliations:** 1 Department of Molecular Biology and Genetics, Çanakkale Onsekiz Mart University, Çanakkale 17100, Türkiye; emirakdasci@stu.comu.edu.tr (E.A.); furkan.eker@stu.comu.edu.tr (F.E.); hatice.duman@comu.edu.tr (H.D.); 2 European Institute for Membranes (IEM)—UMR 5635, University of Montpellier, ENSCM, CNRS, 34090 Montpellier, France

**Keywords:** silver nanoparticles, green synthesis, bio-based synthesis, bacteria-based synthesis, fungi-based synthesis, biomedical applications

## Abstract

The growing demand for sustainable and eco-friendly technologies has driven the development of green and bio-based synthesis methods for metallic nanoparticles. Among these, the microbial synthesis of silver nanoparticles (AgNPs) has emerged as a promising alternative to conventional chemical methods, which often rely on hazardous reagents and harsh conditions. Bacteria and fungi are particularly attractive due to their ability to produce AgNPs with tunable size, shape, and surface properties through natural enzymatic and metabolic processes. This review provides a comparative analysis of bacterial and fungal synthesis routes, focusing on their distinct advantages, limitations, and optimal applications. Bacterial synthesis offers faster growth, simpler culture requirements, and greater potential for genetic manipulation, enabling precise control over nanoparticle (NP) characteristics. In contrast, fungal synthesis typically yields higher nanoparticle stability and is well suited for extracellular, scalable production. The review also summarizes key synthesis parameters (e.g., pH, temperature, reaction time), addresses reproducibility and scalability challenges, and highlights emerging research areas, including antibacterial bio-hybrid materials and bacterial-supported metallic catalysts. Overall, this comparative perspective provides a clear framework for selecting appropriate microbial systems for different technological applications and identifies future research directions to advance green nanotechnology.

## 1. Introduction

The growing need for sustainable and eco-friendly nanotechnologies has resulted in the development of methods that use microbes to produce silver nanoparticles (AgNPs) [1]. Unlike the conventional chemical synthesis methods, which often suffer from toxic reagents and disadvantageous conditions, microbial-based green synthesis offers an environmentally friendly alternative utilizing the natural metabolic and enzymatic processes to generate AgNPs with tunable properties. Among various green sources, bacteria and fungi have emerged as highly promising sources with the ability to produce stable, biocompatible, and reactive NPs [2].

Bacteria-based green synthesis of AgNPs offers an efficient and sustainable alternative to conventional chemical synthesis methods. The diverse biomolecule profile of bacteria, including enzymes and polysaccharides that act as reducing and stabilizing agents, contributes to the eco-friendly nature of this approach. AgNP synthesis can be initiated with various bacteria strains, allowing for a wide range of physicochemical properties, such as size and shape, which are essential for different applications [3]. This flexibility enables the manipulation of AgNP properties and other synthesis parameters, enhancing the versatility of bacteria-based AgNPs. Additionally, AgNP synthesis can occur either intracellularly or extracellularly, with extracellular synthesis being preferred due to the simpler purification process compared to the intracellular method [4].

Fungi-based green synthesis, commonly referred to as mycosynthesis, emerges as one of the mainly utilized green synthesis approaches for the production of AgNPs. It exhibits a mechanism where silver ions (Ag^+^) are reduced to elemental silver (Ag^0^) with the help of fungal biomolecules, including the enzymes, secondary metabolites, and proteins [5]. The mechanism of fungi-based AgNP synthesis is broadly divided into two categories: intracellular and extracellular. Intracellular synthesis is focused on the formation of NPs within the microorganism, which requires further purification steps to obtain AgNPs. Extracellular synthesis, on the other hand, is mostly preferred over intracellular methods, as it enables the simpler production of NPs in a filtrate containing fungal elements without the requirement of further purification [6]. Fungi-based synthesis also steps up in comparison to bacteria-based methods, as it provides multiple benefits, such as ease of cultivation, increased growth rates, and higher metabolite production, making it suitable especially for mass production [7].

Green synthesized AgNPs exhibit significant potential in various applications, mostly in the biomedical, agricultural and environmental areas due to their eco-friendly, highly stable and cost-effective nature. Many studies in the current literature highlight the broad-spectrum utilization of AgNPs in the biomedical area as antimicrobial agents, especially as antibacterial agents due to their immense effectiveness against a wide range of bacteria, including the multidrug-resistant (MDR) strains [1].

AgNPs produced from bacterial and fungal sources also hold potential in anticancer studies, showing effectiveness against various cancer cell lines [8,9,10]. The reason behind this is their capability to increase reactive oxygen species (ROS) formation, inhibit the growth of tumors and trigger apoptosis. Moreover, in addition to their sole utilization, AgNPs can be combined with conventional chemotherapeutic drugs to enhance the treatment efficiency while offering eco-friendly and sustainable alternatives to current strategies [11].

Another area where researchers are exploiting the advantageous traits of the green-synthesized AgNPs is in agricultural studies, where they are used to improve soil quality, stimulate plant growth, promote seed germination and protect crops from plant pathogens, due to their low toxicity and biocompatibility in comparison to agrochemicals [12].

Furthermore, both bacteria- and fungi-mediated approaches yield AgNPs with improved stability, high reactivity, broad-spectrum antimicrobial activity and biocompatibility, which is immensely desired for environmental applications. These applications mostly include water remediation and photocatalysis, where AgNPs efficiently disinfect the wastewater infected with bacteria and their biofilms, as well as accelerating the removal of industrial dyes [13,14].

Considering these, this review provides a comprehensive analysis of the synthesis methods, optimization studies, recent applications, and challenges associated with the AgNPs green synthesized from both bacterial and fungal sources. Regarding the increasing number of publications focusing on the production of AgNPs through eco-friendly routes, this review focuses on recent advancements and current trends that highlight sustainable approaches, underlining the potential of microorganism-based synthesis as a viable alternative to conventional methods. Together with a comprehensive review of the synthesis methods, this review also addressed the most recent scientific advancements in the field of green synthesis of AgNPs and their applications. These NPs are suitable for a diverse range of applications, including antimicrobial therapy, cancer treatment, agriculture, and environmental remediation, due to their exceptional physicochemical and biological properties. This review highlights the significance of microbial synthesis in the advancement of a more sustainable, safer, and environmentally friendly future for nanotechnology by establishing a connection between fundamental research and practical implementation.

## 2. Green Synthesis of AgNPs

Green synthesis approaches are guided by several key principles, including the reduction in or prevention of waste generation, utilization of non-toxic or low-toxic materials, reliance of renewable sources, developing energy-efficient processes, and minimization of secondary byproduct formation [15]. Green synthesis has become an increasingly preferred approach for nanomaterial production in recent years, considering the expanding research focus and application potential of various nanostructures. Conventional synthesis methods often involve hazardous processes with a notable risk for the environment, while green synthesis aims at the elimination of harmful byproducts during the process [16].

**Figure 1:**
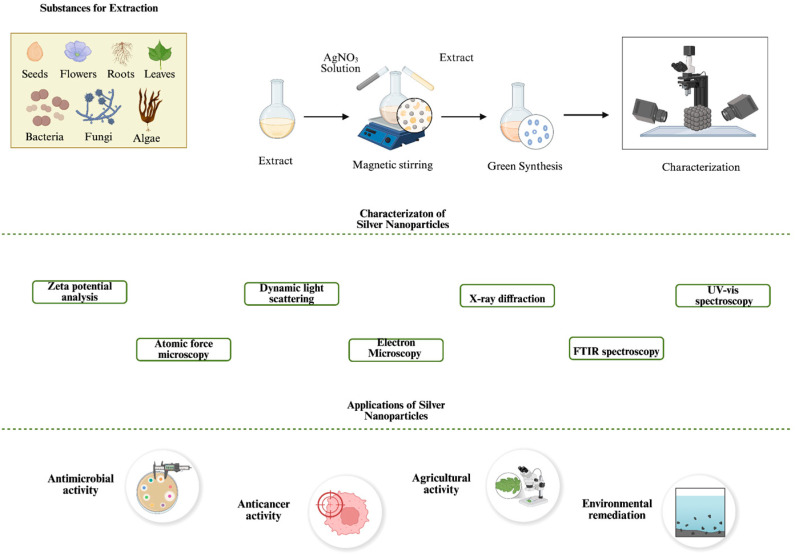
Green Synthesis Mechanism of NPs, and Their Characterization and Applications [2,19].

Several types of renewable sources are highly used in green synthesis of NPs, such as bacteria, fungi, algae and plant extracts (Figure 1). Among these, plant extracts are one of the most preferred sources in green synthesis of AgNPs in the last few years [17]. The availability of diverse plant species and cost-effective procedures further contributes to the widespread application and advantages of plant-based green synthesis. Similarly, microorganisms, specifically fungi and bacteria, are widely employed in NP synthesis. Their use offers the potential of high yields, cost-effective production (though not in all cases), and tunable physicochemical properties when synthesis conditions and environmental parameters are properly optimized [18].

Still, current optimizations regarding microorganism-based NP synthesis are not sufficient compared to more practical approaches of plant-based synthesis. For instance, while the reduction process using plant-based reducing agents may initiate within a few hours and could complete the process within 48 h, the reduction process mediated by microorganisms often takes at least three times longer [20]. Despite this extended reaction time, microorganism-based synthesis offers significant potential due to their rich biochemical composition, including enzymes, proteins, and sugars that can be actively involved in the reduction and stabilization of AgNPs.

Despite their significant potential, green synthesis of NPs has several challenges when considered for wide-ranging applications. They exhibit distinct advantages and limitations based on the biological source (Table 1). These include limited control over key physicochemical properties, particularly size and shape, difficulty in achieving high yields, challenges in selecting the most suitable biological source, and a lack of comprehensive optimization strategies; all of these hinder the broader application of green synthesis in NP technology [21].

Nevertheless, the significant potential of green synthesis has a growing attraction with the increasing demand for sustainable nanomaterials. As a result, a significant number of studies have discussed and analyzed the utilization of these eco-friendly routes across diverse biological platforms.

Antimicrobial coatings represent one of the most extensively studied applications of nanomaterials. Considering their strong antibacterial properties, AgNP-based coatings hold significant potential in this area. A particularly promising application of green-synthesized nanomaterials is the development of biodegradable food packaging materials [28]. The incorporation of NPs into food materials can enhance mechanical properties, improve physicochemical stability, and reduce microbial contamination through their combined antioxidant and antibacterial activities [29]. Given the increasing demand for sustainable and non-toxic packaging alternatives, green synthesis offers a particularly attractive route for producing AgNPs for food-related applications. Similarly, applications focusing on the environmental aspect, such as water treatment and dye degradation, represent key areas for the utilization of green synthesized nanomaterials. Due to their catalytic properties and strong antibacterial characteristics, green synthesized AgNPs have attracted significant attention in these fields. For instance, plant-based AgNPs have emerged as an effective alternative to conventional chemical disinfectants for water purification [30]. In addition, green synthesized AgNPs are known to be effective in removing excessive toxic heavy metal ions from aqueous systems and catalyzing degradation of various dyes. These multifunctional properties make green-synthesized AgNPs as promising agents in environmentally sustainable technologies. Even further, Larrañaga Tapia et al. demonstrate that green-synthesized bimetallic NPs are recognized as potential tools that align with United Nations Sustainable Development Goals, including water purification and responsible consumption, through combination of incorporation of green nanotechnology [31]. Thus, green-synthesized AgNPs align well with upcoming sustainability goals, making them alternatives for the development of antimicrobial and catalytic nanomaterials.

**Figure 2:**
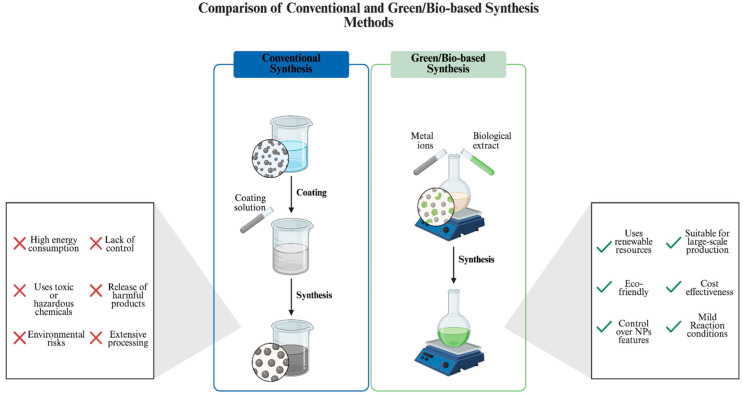
Comparison of Conventional and Green/Bio-based Synthesis Methods [37].

Collectively, there is an abundance of research focusing on the green and sustainable synthesis of various materials, particularly NPs, with emphasis on their characterization and potential applications [32,33,34,35,36]. This expanding research significantly contributes to addressing existing gaps in synthesis protocols by offering insights into the control of physicochemical properties, paving the way for more standardized and scalable methods. A green synthesis approach will benefit from such a background for environmental and industrial applicability. Furthermore, it can promote the development of interdisciplinary approaches, where all biological, chemical and engineering perspectives can be integrated to support the applications of green synthesized NPs across a wide range of fields. Considering these factors, a detailed investigation of green synthesis, particularly focusing on well-established biological sources such as bacteria and fungi, offers critical insights for further optimizing green-based NP production (Figure 2).

## 3. Bacteria-Based Green Synthesis of AgNPs

Bacteria is one of the most efficient agents that is utilized in the green synthesis of NPs, particularly in AgNP synthesis. Several biomolecules that are produced from bacteria, such as enzymes, proteins and polysaccharides, can act as reducing agents to facilitate production of NPs [38]. Bacteria-mediated synthesis of AgNPs represents a sustainable and environmentally friendly alternative to conventional chemical and physical methods. This biosynthetic method removes the need of toxic reagents and high-cost processes, thereby paving the way of eco-friendly NP production and application [39].

### 3.1. Mechanism of Bacterial Reduction of Silver Ions

The general mechanism of bacteria-mediated AgNP synthesis involves the biological reduction of Ag^+^ to Ag^0^. This process is primarily driven by the metabolic activity of bacterial cells, which facilitate the reduction either through enzymatic action or with the secretion of bioactive bacterial metabolites.

Bacteria-mediated synthesis of AgNPs can occur through either intracellular or extracellular pathways. While intracellular synthesis involves ion trapping and reduction within the cell, extracellular synthesis relies on secreted biomolecules and is generally preferred due to its simpler handling and downstream processing [38,40].

Several key biological agents are involved in the reduction of Ag^+^ during the NP synthesis. Among these, enzymes derived from bacteria, particularly reductase enzymes such as NADH-dependent reductases, have been proposed to play a central role by donating electrons to facilitate the bioreduction of Ag^+^ to Ag^0^ [41]. These enzymes can operate in both intracellular and extracellular environments, which may depend on the bacterial species and the localization of Ag^+^.

Loi et al. synthesized AgNPs from nitrate reductase produced by *Lactobacillus plantarum* CAM 4 [42]. The authors optimized the lactic acid bacteria fermentation medium for nitrate reductase production, which was effective as the characterization of AgNPs confirms. Moreover, the synthesized AgNPs exhibited notable antifungal and antibacterial activity, demonstrating their antimicrobial potential along the study. Saba et al. utilized keratinase from *Pseudomonas aeruginosa* (*P. aeruginosa*)-C1M for the green synthesis of AgNPs, which exhibited strong antibacterial and catalytic activity [43]. The AgNPs demonstrated effective antibacterial activity against commonly tested bacterial strains, *Escherichia coli* (*E. coli*) and *Staphylococcus aureus* (*S. aureus*), and showed notable catalytic degradation of azo dyes such as safranin o and methyl orange.

Although several studies have investigated the interaction between bacterial enzymes, particularly reductases [44,45], and Ag^+^, recent research specifically exploring the isolated utilization of these enzymes in green synthesis remains notably limited.

Microbial metabolites can also initiate the reduction of Ag^+^. Diverse types of bacterial biomolecules, such as proteins, exopolysaccharides and phenols, are known to facilitate bioreduction of metal ions, including Ag^+^ [46].

Plokhovska et al. synthesized AgNPs from metabolites of *Pseudomonas* sp. N5.12, which functioned as both reducing and coating agents during the synthesis process [47]. The resulting small, spherical AgNPs, ranging between 13.75 ± 0.47 nm to 20.71 ± 0.43 nm, exhibited strong antimicrobial activity against various fungal and bacterial species. Additionally, these green-synthesized AgNPs were effective against phytopathogens, highlighting their potential for applications in the agricultural sector. Similarly, Amal et al. green-synthesized AgNPs using secondary metabolites from *Rhodococcus rhodochrous* (MOSEL-ME29) and *Streptomyces* sp. (MOSEL-ME28), and evaluated their biological activities [48]. Both types of AgNPs demonstrated antibacterial activity, although their zone of inhibition (ZOI) was lower compared to the control antibiotic group (Gentamycin). In addition to antibacterial testing, the AgNPs were tested for antileishmanial, anticancer, antiviral and antioxidant activities. The study highlights the potential of marine actinobacteria-derived AgNPs for a broad range of biomedical applications.

Finally, functional groups associated with bacterial cells, including those located on the cell wall, are also believed to contribute to the reduction of Ag^+^. Functional groups present in metabolites of bacteria, such as carboxyl, hydroxyl and phosphate, can effectively initiate the Ag^+^ reduction [49]. As an example, Sambalova et al. investigated the reduction of Ag^+^ and their stabilization in isolated biofilm matrices [50]. Within the investigation, they have reported that the majority of the interactions between the AgNPs and biopolymers consisted of carboxylate functional groups. Characterization using SERS confirmed the consistent presence of carboxylate functional groups on the NP surface, contributing to understanding of the interactions between microbial components and AgNPs.

Several studies have highlighted the involvement of these groups in bacteria-based green synthesis of AgNPs. Kwon et al. synthesized AgNPs using *Aggregatimonas sangjinii* F202Z8T, demonstrating their antibacterial efficiency [51]. The FTIR analysis revealed the functional groups that are potentially involved in the synthesis process, serving as capping agents to stabilize the particles. Similarly Adebayo-Tayo et al., synthesized AgNPs from *Oscillatoria* sp. extract and reported their antibacterial, antibiofilm and cytotoxicity activities [52]. They also noted the contribution of functional groups, involving hydroxyl and amine groups, during the synthesis as capping agents and particle stabilization.

### 3.2. Effect of Synthesis Parameters on Physicochemical Properties and Biological Activities of AgNPs

The physicochemical characteristics of AgNPs are strongly influenced by the synthesis conditions. In bacteria-mediated synthesis, parameters such as pH, temperature, Ag^+^ concentration, bacteria strain, and metabolic profile can significantly affect the properties of the produced NPs, thereby influencing their biological activity [38].

A study demonstrated the influence of bacterial strain on the physicochemical properties of synthesized AgNPs using four different *Geobacillus* spp. [53]. Certain characteristics, such as the surface plasmon resonance (SPR) peak (ranging between 410–425 nm) and zeta potential (ranging from −25.7 ± 0.8 to −31.3 ± 0.8 mV), were found with small differences across samples. Similarly, all types of AgNPs exhibited a spherical morphology. However, there was a notable difference in the average particle size among the bacterial strains. While 25% of the AgNPs synthesized by strain 95 had sizes greater than 100 nm, only 1% of the AgNPs synthesized from strain 25 had such high particle size. This highlights the broad size distribution influenced by the bacterial strain used in the synthesis process.

Another study synthesized AgNPs using three different types of bacteria and their intracellular extract and supernatant, separately: *Cupriavidus necator*, *Bacillus megaterium*, and *Bacillus subtilis* (*B. subtilis*) [54]. The authors investigated the effects of pH and temperature on the AgNP synthesis, emphasizing their influence on both the physicochemical properties and the biological activity of AgNPs, in addition to the type of bacterial source used. Depending on the source of stabilizing and capping agents, either from intracellular extract or supernatant, each type of AgNP demonstrated different zeta potential and particle size. For instance, AgNPs synthesized from the extracellular extract of *B. subtilis* exhibited an average hydrodynamic diameter of 118.4 ± 1.9 nm, whereas those synthesized from supernatant measured 20.8 ± 0.6. Similar variations in zeta potential values were observed in AgNPs synthesized from *B. subtilis* and *B. megaterium*, highlighting the effect of bacteria species and their source. The study also reported distinct SPR peaks depending on the pH and temperature, further demonstrating the sensitivity of AgNP properties to synthesis conditions. While all AgNPs maintain predominantly spherical morphology, their antibacterial activities are also greatly affected. Among the AgNPs synthesized from supernatants, *B. megaterium* showed the highest antibacterial activity against *E. coli* with minimum inhibitory concentration (MIC) value of 2.750 ± 0.487. On the contrary, AgNPs derived from the intracellular extract of *C. necator* demonstrated the highest antibacterial activity with MIC value of 8.800 ± 1.230, whereas those from *B. megaterium* showed the lowest activity.

Sharma et al. utilized various probiotic strains, *Lactobacillus pentosus* (*L. pentosus*) S6, *Lactobacillus plantarum* (*L. plantarum*) F22, *Lactobacillus crustorum* (*L. crustorum*) F11, and *Lactobacillus paraplantarum* (*L. paraplantarum*) KM1, for the green-synthesis of AgNPs to evaluate their antimicrobial activity against MDR pathogens [55]. Although the absorbance values varied among the particles, all AgNPs exhibited a characteristic SPR peak at 450 nm and shared a predominantly spherical morphology However, notable differences in average particle size were observed in comparison: AgNPs synthesized from *L. pentosus* S6 and *L. paraplantarum* KM1 exhibited average size of 50 nm, whereas those from *L. plantarum* F22 and *L. crustorum* F11 had average size of 20 nm and 10 nm, respectively. These differences are followed by their antimicrobial efficiency, as AgNPs synthesized from *L. crustorum* F11 showed the highest antibacterial activity against all tested strains, with a ZOI reaching up to 20 ± 0.61 mm. Similarly, in antifungal assays, AgNPs synthesized from *L. crustorum* F11 suppressed all other samples with the largest ZOI recorded at 32.6 ± 0.48 mm.

Another study performed extracellular synthesis of AgNPs using *Leclercia adecarboxylata* THHM, demonstrating their optimization and antimicrobial activity [56]. During the experimental process, several synthesis parameters were evaluated to optimize the green synthesis of AgNPs. Initially, no significant AgNP formation was observed within the first 24 h of incubation. However, a notable increase in AgNP formation was observed up to 48 h, where the colloidal solution exhibited the highest stability. AgNO_3_ concentration was directly proportional with the AgNP yield, although authors selected 1.0 mM as the optimum concentration to maintain chemical stability and prevent agglomeration. Regarding pH levels, AgNP production improved effectively between pH 5 and 7, peaked at pH 7, but declined significantly at higher pH levels, reaching the lowest production rate at pH 10. Temperature also played a crucial role, with the highest AgNP production observed at 40 °C, followed by a slight decrease at 45 °C. Unlike AgNO_3_ concentration, volume of *Leclercia adecarboxylata* THHM supernatant showed an inverse effect, demonstrating optimal production at 20% (*v*/*v*), while having the lowest yield at 100% (*v*/*v*). Finally, Plackett–Burman design (PBD) analysis revealed that pH was the only parameter that negatively influenced the AgNP synthesis. The green-synthesized AgNPs exhibited powerful antimicrobial activity against multiple clinical pathogen strains.

These results clearly demonstrate that not only synthesis conditions significantly alter the physicochemical properties of AgNPs, but the bacterial source of capping and stabilizing agents also plays a crucial role in determining their biological activity. Given the significant differences observed among the synthesized AgNPs, it is essential to systematically identify which synthesis parameters influence specific attributes, and to optimize these parameters accordingly for effective performance. Despite the growing interest in microbial-based synthesis of AgNPs, direct investigations examining how specific bacterial strains or their metabolites influence the physicochemical properties of NPs remain significantly limited. While some studies have indirectly highlighted strain or metabolite-dependent differences in AgNP size and shape, these are often isolated findings rather than part of a systematic framework. Comparative analyses are mostly restricted to a few cases where similar bacterial strains or metabolites were used under controlled conditions, allowing for some degree of inference. However, a comprehensive and standardized comparison of how different bacterial metabolites modulate nucleation, growth, and stabilization processes, and consequently NP morphology and size distribution, is still insufficient in the current literature.

### 3.3. Advantages and Limitations of Bacteria-Mediated AgNP Synthesis

As with most forms of green synthesis, bacteria-mediated NP synthesis presents featured advantages, along with certain limitations. In general, alongside the common advantages shared with other green synthesis approaches, such as low energy consumption, the absence of toxic chemicals, and utilization of renewable resources, bacteria-based synthesis often produces NPs with smaller size and enhanced stability [2]. Furthermore, certain bacterial strains contribute to the production of high-quality NPs. For instance, given their ability to grow and survive under extreme environmental conditions, extremophilic bacteria represent a unique alternative within bacteria-based NP synthesis. These organisms can produce reducing and capping agents that remain stable and highly resistant under harsh conditions, leading to the formation of NPs with enhanced chemical stability and resistance [57].

The source of capping and stabilizing agents from bacteria, which initiates AgNP synthesis and strongly influences NP quality, is an important factor. As discussed, the biological activity of AgNPs can vary significantly depending on whether they are synthesized intracellularly or extracellularly. This difference also introduces additional challenges in isolating these NPs, requiring additional steps. Isolation of AgNPs from the intracellular environment requires additional procedures, including usage of chemicals with sonication and autoclaving, while that is much simpler after the extracellular synthesis of the particles [58].

Moreover, some key challenges should be addressed to facilitate the bacteria-mediated synthesis of AgNPs for wide-ranging applications. Some of the key problems within this approach is the possibility of contamination, inefficient length for certain procedures, long incubation times and less flexibility over controlling the particle size during the synthesis [59]. Moreover, depending on the strain, bacteria-based synthesis possesses lower metal tolerance and lesser amounts of particle production, specifically when compared to fungi-based synthesis [24].

To address such limitations and expand the versatility of green synthesis approaches, other biological systems should be explored. For instance, algae-based synthesis offers a significant advantage by enabling NP synthesis under a wide range of conditions, reducing cultivation costs, and opening opportunities for bioremediation applications [60]. On the other hand, fungal-based synthesis offers several advantages that enhance its potential for large-scale applications, including its ability to produce high amounts of extracellular enzymes, efficient metal absorption, and growth capability on thin-layer materials [61].

Bacteria-mediated green synthesis of AgNPs presents a promising alternative to conventional synthesis methods. By leveraging the advantages of green synthesis, this approach offers an eco-friendly, sustainable and cost-effective pathway for NP production. Similarly to other biological methods, synthesis conditions can be designed to produce NPs with specific physicochemical characteristics. However, further development is needed through comprehensive optimization strategies. One of the most notable benefits of bacteria-based synthesis is its ability to facilitate extracellular enzyme-mediated reduction and stabilization of AgNPs, which simplifies the recovery process and enhances particle stability. To address the existing challenges, a broader investigation into other green synthesis methods, such as fungal and algal systems, may offer essential insights for all types of approaches. Also, a deeper understanding of the enzymatic mechanism involved in Ag^+^ reduction is essential in general. Furthermore, limitations in standardization and certain complexities associated with bacterial systems must be addressed to enable reliable large-scale production. Addressing these challenges, along with precise selection of bacteria strains and synthesis parameters, is crucial for unlocking the full potential of bacteria-mediated AgNP synthesis in industrial and biomedical applications.

## 4. Fungi-Based Green Synthesis of AgNPs

In addition to bacteria-mediated synthesis, fungi-directed methods have been widely explored for the synthesis of AgNPs as an eco-friendly, biocompatible and reproducible alternative. Since biological systems serve as natural reducing, stabilizing and capping agents, they eliminate the use of excess chemicals, which prevents toxicity and enables the production of large quantities of NPs with high yields [62]. Specifically, fungi confer unique advantages owing to their ability of secreting wide-ranging enzymes, metabolites and proteins involved in AgNP formation. Diversity among fungal species also contribute to the production of AgNPs with different physicochemical properties, including controlled size, uniform morphology and high monodispersibility [63].

### 4.1. Mechanism of Fungal Reduction of Silver Ions

Similarly to bacteria-mediated synthesis, fungi-mediated synthesis relies upon the mechanism of the reduction of Ag^+^ to Ag^0^. The process of the conversion of Ag^+^ to Ag^0^ is facilitated by fungal biomolecules, such as proteins, polysaccharides, secondary metabolites and enzymes. In intracellular synthesis, AgNPs are produced within the microorganism, where metal precursor binds to the proteins or enzymes present on the cell wall of fungi due to electrostatic interactions. Then NPs are extracted through centrifugation, chemical treatment and filtration. Whereas in extracellular synthesis, the metal precursor, such as AgNO_3_, is included in the aqueous filtrate containing various fungal cellular constituents including proteins, enzymes, peptides and amino acids, which carries out the synthesis of AgNPs [40,63].

Intracellular synthesis methods have been known to yield AgNPs with relatively larger sizes and high monodispersibility; yet, they require more complex and labor-intensive purification steps to obtain NPs. Extracellular methods, on the other hand, are commonly preferred due to their simplicity, cost-effectiveness and ease of further processing as they eliminate the need for breaking down the fungal cells [5].

Recently, several fungal species such as *Fusarium oxysporum* (*F. oxysporum*), *Aspergillus niger* (*A. niger*), and *Penicillium* spp. have exhibited notable capabilities in the reduction of Ag^+^ to Ag^0^ [64,65,66]. In addition, similar to bacteria, these fungi are capable of producing a wide range of biomolecules such as NADH-dependent reductases, nitrate reductase, quinones and other redox-active compounds that not only facilitate the production of AgNPs but also play a key role in the stabilization of the resulting NPs by capping them and preventing their aggregation [67].

Accordingly, Ali et al. synthesized AgNPs extracellularly from *Fusarium equiseti*, which is known for its powerful metabolic capacity and to release extracellular enzymes, particularly NADH-dependent nitrate reductase, polysaccharides, phenolic compounds and proteins that will enhance the NP stability and biological activity. To maximize the enzyme production researchers cultured *F. equiseti* in Sabouraud Dextrose Broth (SDB) at at 28 ± 2 °C under shaking conditions, 150 rpm, for 5 days. Further, AgNO_3_ was included into the filtrate of the growth culture, obtaining a final concentration of 1 mM, and synthesis proceeded in the dark under static conditions to enable the NP synthesis. Additionally, synthesized AgNPs exhibited antimicrobial activity against various bacterial and fungal pathogens, as well as antioxidant and cytotoxic activity [68].

In another study, Lotfy et al. utilized *Aspergillus terreus* BA6 for the eco-friendly synthesis of AgNPs. To promote secretion of nitrate reductase, they inoculated 1 mL of spore suspension in SDB medium and proceeded incubation at 25 °C under shaking conditions, 200 rpm, for 5 days, achieving approximately 20 g of fungal mycelia after the filtration. Similarly, they added 200 μL of 0.5 M AgNO_3_ to 100 mL of filtrate to obtain a final concentration of 1 mM of AgNO_3_, facilitating the successful production of NPs under the same conditions for 2 days. Researchers also revealed antimicrobial activity of myco-synthesized AgNPs against 12 reference bacterial and fungal pathogens, as well as demonstrating their significant antitumor efficiency. However, AgNPs were not able to inhibit infectivity of Coxsackie B virus infected Vero cells [69].

Apart from the enzymatic contribution, Kaur et al. highlighted the role of biomolecules found in fungal extract to produce AgNPs. In their study *Penicillium camemberti* MTCC 418 was cultured in Potato Dextrose Broth (PDB) at 28 °C for 96 h to obtain the fungal filtrate. FT-IR analysis confirmed the presence of a high amount of amino acids, proteins and heterocyclic compounds that are primarily associated with the reduction in Ag^+^ and stabilization of the resulting NPs. Further AgNPs synthesis was carried out in 25 °C, which was identified as the optimal temperature for the activity and stability of the enzymes found in fungal extract. Concerning the pH, researchers determined pH 7 to be the most effective, while highly basic or acidic conditions led to disruption of reductase activity and thereby to unsuccessful NP synthesis. The resulting AgNPs demonstrated antibacterial, antioxidant, anti-inflammatory and anti-diabetic activity, highlighting the promising potential of the eco-friendly synthesis of AgNPs from fungal sources [70].

Overall, the mechanism behind fungi-mediated synthesis is influenced by a combination of enzymatic and non-enzymatic biomolecules secreted during the fungal growth. For instance, the abundance of extracellular enzymes, including NADH-dependent reductases or nitrate reductase, as well as proteins, amino acids and diverse secondary metabolites contribute to the successful reduction of Ag^+^ to Ag^0^ and stabilization of the formed NPs. However, it is important to mention that the efficiency of NP synthesis is highly dependent on fungal species used, cultivation conditions, and composition of the filtrate. Hence, understanding these processes and optimizing the key synthesis parameters hold great significance to maximize the functionality and applicability of the myco-synthesized AgNPs.

### 4.2. Effect of Synthesis Parameters on Physicochemical Properties and Biological Activities of AgNPs

Physicochemical properties like size, shape, and surface functionalization consequently influence the biological functions of NPs, such as antimicrobial and anticancer activity through the modulation of their interaction with microbial membranes and cellular targets [63]. Yet, to achieve these favorable properties, many variables including the AgNO_3_ concentration, pH, and temperature of the environment must be carefully controlled. Optimizing these parameters is crucial for synthesizing NPs with the desired size, shape and traits, which can be tailored for specific applications. Accordingly, many studies are present in the current literature for exploring the influence of these factors on the morphology, stability and functionality of NPs [5].

For example, Saxena et al. highlighted the effect of growth medium, biomass, AgNO_3_ concentration, pH and temperature on the extracellular green synthesis and physicochemical properties of AgNPs derived from plant pathogenic fungi *Sclerotinia sclerotiorum* MTCC 8785. They have utilized a wide range of culture media, including PDB, SDB, Czapek Dox (CZAPEK), Glucose Yeast Extract Peptone (GYP), Protease production media (PP) and Richard medium (RM). Among these, fungal biomass that were grown in PDB have shown enhanced AgNPs synthesis, followed by SB, RM, CZAPEK, PP and GYK. This is attributed to the presence of ingredients in PDB that stimulate better fungal growth and facilitate the production of increased levels of reducing agents. In addition, extracellular synthesis of AgNPs were conducted using varying amounts of fungal biomass, ranging between 0.5 and 10 g. It was found that when the amount of biomass was increased, synthesis of AgNPs was also increased. Optimum pH of the environment was determined as 11, supported by the maximum AgNP production compared to pH 3, 5, 7 and 9. They have also investigated the effect of AgNO_3_ concentration (ranging from 0.2 to 2 mM) and temperature (from 20 to 80 °C) on NP production. Results revealed increased concentration of AgNO_3_, up to 2 mM, enabled the complete reduction of Ag^+^ ions. Additionally, the maximum and most stabilized synthesis of AgNPs were observed at 80 °C, at pH 11 and in the presence of 2 mM AgNO_3_ concentration. NPs produced under these conditions further demonstrated antibacterial activity against *E. coli* and *S. aureus*, at a concentration of 100 ppm, with a particular zone of inhibition of 20 mm against *E. coli* [71].

Osorio-Echavarría et al. focused on the influence of fungus growth time and reduction method AgNO_3_ concentration on the green synthesis of AgNPs from white-rot fungus *Anamorphous bjerkandera* sp. R1. Apart from general approaches, they have utilized two different reduction methods, one including the reduction of Ag^+^ from fungal filtrate (CS) and the other reduction of Ag^+^ from the mycelium-pellets (MP). To determine the effect of AgNO_3_, they have evaluated three different concentrations, 0.5, 1.0 and 1.5 mM. To evaluate the optimum growth time for fungus, six different time periods, 3, 4, 5, 6, 7 and 8 days were assessed. Optimum conditions were determined as 8 days of fungus growth time, 144 h of reaction time under 30 °C and 1 mM of AgNO_3_ concentration. Under optimal conditions CS samples achieved spherical NPs with size ranging from 10 to 30 nm, with high SPR peaks at 430 nm. In contrast, MP samples led to production of larger NPs, ranging between 70 and 100 nm with spherical morphology [72].

Nayak et al. investigated the effect of AgNO_3_ concentration and pH of the reaction medium for the green synthesis of AgNPs using *Penicillium purpurogenum* NPMF fungi. Results revealed increased AgNP production through increased AgNO_3_ concentration, up to 1.5 mM, following 24 h of incubation. Varying AgNO_3_ concentrations were also found to influence the size and shape of the NPs. At 1 mM, highly monodispersed spherical NPs with an average size of 10 nm, along with smaller NPs having 4 to 6 nm sizes, were observed. In contrast, 3 mM of AgNO_3_ concentration led to the production of polydispersed star-shaped NPs, ranging in size between 5 and 40 nm. Concerning the 5 mM of AgNO_3_ concentration, unevenly shaped, polydispersed NP production was observed. Starting with an initial pH of 8.3, researchers also observed the effect of varying pH levels on the formation of AgNPs. It was stated that different pH levels significantly affected the morphology and size of the NPs. Larger NPs, with average particle size between 40 to 55 nm, having many different shapes such as spherical, ellipsoidal and pyramidal were formed at pH 4 and 5. However, at pH 8 and 9, more uniform and smaller NPs, with sizes between 8 and 13 nm, with mostly spherical shape were formed. Antimicrobial activity of synthesized AgNPs further evaluated against both Gram-positive (*S. aureus*) and Gram-negative (*E. coli* and *P. aeruginosa*) bacteria. An increase in AgNO_3_ concentration, from 0.50 mm to 5 mm, led to notable improvements in bactericidal efficiency, while NPs synthesized using 0.25 mM AgNO_3_ showed no activity. Conversely, different pH conditions resulted in variable outcomes, with the minimum zone of inhibition observed at pH 6. This is attributed to the differences in shape and surface area of the synthesized AgNPs, with being more potent against Gram-negative strains, as the authors stated [73].

In another study, Soleimani et al. assessed the effect of pH and temperature for the synthesis of AgNPs from four different fungi, including three isolates of *Beauveria bassiana* (*B. bassiana*) (JS1, JS2 and KA75) and one isolate of *Metarhizium anisopliae* (*M. anisopliae*) (TT1). In order to determine the optimum conditions two different temperatures (40 and 60 °C) and four different pH levels (5, 6, 7 and 8) were evaluated. At pH 7 and 60 °C, researchers highlighted the highest production rates, despite a reduction in AgNPs yield noted at pH 5 under the same temperature. At optimal conditions, the smallest AgNPs with sizes ranging from 23.3 nm to 76.51 nm (JS1, JS2 and KA75) and average diameter of 101.34 nm (TT1) were obtained. Conversely, pH 8 produced the largest AgNPs regardless of the temperature. Through antimicrobial assays, researchers revealed that the smaller AgNPs had better antifungal activity against *B. bassiana*, *M. anisopliae* and plant-pathogenic *Rhizoctonia solani*, achieving complete inhibition of JS2 fungus with TT1-AgNPs at a concentration of 15 µL/mL [74].

Zhu et al. synthesized AgNPs from *Penicillium polonicum* focusing on AgNO_3_ concentration (5, 10, 20, 40, 80 and 160 mM), temperature (4, 28, 37, 45 °C), pH (4, 5, 6, 7, 8, 9, and 10) and incubation time (12, 24, 48, and 60 h). They identified the key conditions for NP synthesis as 80 mM of AgNO_3_, 48 h of incubation, 45 °C and pH 9, which produced NPs within the size range of 3 to 25 nm and spherical morphology. In more detail, they have shown that increasing pH up to 11.0, led to corresponding increases in particle size. Concerning the temperature, the highest yield and most efficient reduction of filtrate was observed at 45 °C. When reaction time is investigated, a maximum absorption peak of 450 nm was observed at 60 h, however a shift occurred when it decreased down to 48 h, indicating a decrease in particle size [75].

Smirnov et al. utilized *Ganoderma lucidum*, also known as Reishi mushroom, extracts with different pH values to synthesize AgNPs. Comparing environments having different pH values, 2.5, 5, 7, 9 and 11, they have confirmed the synthesis of NPs within the range of 10 to 40 nm. The pH variations resulted in distinct localized surface plasmon resonance (LSPR) peak bands, with pH of 2.5 having remarkably smaller intensity, confirming its unsuitability for the NP synthesis. The best LSPR peaks were observed at pH 7 and 9, with pH 11 demonstrating the same sharpness but half the intensity. Also, they revealed the long shoulder of LSPR towards the near-infrared region at pH 5. Following DLS measurements, authors concluded that variation in pH does not affect the average NP size significantly [76].

Apart from the experimental approaches, there are many studies in the current literature highlighting the use of statistical modelling, particularly response surface methodology (RSM) such as Box–Behnken design, to determine optimal synthesis conditions while minimizing the need for extensive experimental procedures. In this way, researchers become able to enhance the synthesis of AgNPs by achieving higher yields and improved control over physicochemical properties [77]. Accordingly, Hoseini-Nilaki et al. synthesized AgNPs from *Alternaria* sp. OP242500. Through the experiments, they have optimized silver acetate concentration, temperature, pH and incubation time, identifying the optimal conditions as 5.5 mM silver acetate, 33.5 °C, pH 7.8 and 96 h of incubation time using the Box–Behnken design. The resulting AgNPs had spherical morphology and face-centered cubic structures, with sizes ranging from 10.9 nm to 68.5 nm. They also demonstrated high stability of NPs with a zeta potential of −20.8 mV [78].

Similarly, Sarkar et al. utilized central composite design (CCD) to improve morpho-physiological characteristics of AgNPs green-synthesized from harvested mushroom extract (HMS). Investigating the effect of pH, temperature and the incubation period, they determined the ideal concentrations as 59.4 °C, pH 8.9 and 48.5 h of incubation. Authors stated that different synthesis conditions led to the formation of NPs of different sizes, with different pH levels 5, 7 and 9 producing NPs having an average diameter of 75 ± 16 nm, 45 ± 12 nm and 35 ± 6 nm, respectively, following 72 h of reaction time at 60 °C. Further, they have revealed that AgNPs predominantly exhibited spherical shapes, with pH values higher than 9 yielding better morphology of NPs [79].

From another perspective, Rose et al. conducted statistical modelling to optimize the production of nitrate reductase in *Aspergillus terreus* N4, an enzyme that facilitates the reduction of Ag^+^ to Ag^0^ for the green synthesis of AgNPs. They optimized the fungal growth medium to achieve the highest production of nitrate reductase, determining the optimal conditions as 1.056% glucose, 1.836% peptone, 0.3386% yeast extract and 0.025% KNO_3_. The synthesis of AgNPs occurred at varying temperatures, 30, 40 and 50 °C, with 40 and 50 °C achieving higher yields and producing smaller NPs during shorter incubation periods. Also, AgNPs synthesized at higher pH levels, 8.0 and 9.0, had the greatest production rates, along with smaller particle size and improved stability, followed by pH 7. Conversely, no particles formed in pH 5 and only slightly formed at pH 6. Regarding the AgNO_3_ concentration, authors determined the highest production at a concentration of 1.5 mM, with lower concentrations (1 mM) being insufficient to produce AgNPs and higher concentrations (2 mM) leading to formation of non-uniformly sized particles and agglomeration. Further, the antimicrobial activity of AgNPs was tested against common foodborne pathogens such as *Salmonella typhimurium* (*S. typhimurium*) and *S. aureus*, creating inhibition zones of 13 mm and 12 mm, respectively, at a concentration of 85 µg/mL [80].

Building on these observations, RSM approaches such as the Box–Behnken design and CCD have become increasingly important in microbial-mediated AgNP synthesis. In fungal systems, these methods have been applied more comprehensively to capture the complex interactions among multiple physicochemical and nutritional parameters. To exemplify, studies on *Penicillium citrinum* (examining AgNO_3_ concentration, solution pH, shaker incubator temperature, agitation rate, and incubation time) and *Trichoderma viride* ATCC 36838 (considering reaction time, AgNO_3_ concentration, percentage of cell-free filtrate, and reaction pH) demonstrated that RSM-based optimization provides higher predictive accuracy, improved NP uniformity, and enhanced control over particle morphology, size, and yield [77,81] This extensive integration of statistical modeling reflects the greater metabolic diversity and enzymatic complexity of fungi, which require the simultaneous consideration of multiple factors influencing NP synthesis. In contrast, bacterial systems have employed RSM and related models less frequently and typically for a smaller number of variables. Although several bacterial studies are available in the literature [82,83], these efforts generally concentrate on a limited set of variables, indicating a more limited application of multivariate optimization compared to fungi [84,85].

In summary, synthesis parameters such as AgNO_3_ concentration, pH, temperature and incubation time collectively influence the physicochemical characteristics and biological activities of the produced AgNPs. Refining these conditions not only enables control over NP size, shape and stability but also improves their antimicrobial activity. In addition, integrating statistical modeling approaches allows for more precise optimization of these synthesis conditions, which facilitate the tailoring of AgNPs to meet specific requirements in diverse application areas while minimizing the experimental procedures. Although fungal-based synthesis of AgNPs has been widely studied, there are still limited investigations that directly examine how specific fungal strains or their metabolites affect the physicochemical properties of the NPs. Most existing studies mention that fungal products contribute to the NP formation but do not clearly explain which specific compounds are responsible for controlling NP size, shape, or distribution. Comparative studies that test different fungal strains or metabolites under similar conditions are especially limited, making it difficult to construct a clear comparison. In contrast, plant-based synthesis studies often include such comparisons, showing how individual compounds influence NP characteristics. This highlights an important gap in fungal-based synthesis research and the need for more focused and comparative studies to better understand the role of fungal metabolites in shaping AgNP properties.

### 4.3. Advantages and Limitations of Fungi-Mediated Synthesis

Fungi-mediated synthesis offers an eco-friendly, sustainable and cost-effective approach over conventional physical and chemical synthesis methods, exploiting the natural characteristics of green synthesis [6]. It minimizes the use of excess chemicals and addresses toxicity risks, while improving the biocompatibility of the resulting NPs [86]. Moreover, current literature demonstrates the enhanced biological activity of AgNPs synthesized via green synthesis routes, with higher antibacterial effectivity and improved control over their physicochemical properties [87].

Fungi-mediated synthesis has also been regarded as a promising strategy among different biological methods used for AgNP synthesis, as it provides greater diversity and higher control over NP size, which is crucial for tailoring their further use [6]. For example when compared to bacteria, previous studies highlight the several advantages of fungi-based synthesis for large-scale production, such as higher growth rates, simplicity in culturing, and increased secretion of extracellular enzymes [7]. The main difference is that fungi generate higher biomass and release higher concentrations of metabolites, which facilitate efficient extracellular NP synthesis. Conversely, bacteria-based methods predominantly depend on intracellular pathways and secrete fewer extracellular enzymes, leading to lower AgNP yields and stability [68].

From another perspective, AgNPs green synthesized from fungal sources can be utilized to combat antibiotic resistance, which is regarded as one of the most major global health challenges. Mycosynthesized AgNPs can inhibit the proliferation of antibiotic resistant bacterial and fungal strains, or produce better results when used in combination with conventional antibiotics [88]. Accordingly, Ibrahim et al. focused on the activity of AgNPs green synthesized from *Candida parapsilosis* against MDR strains of both Gram-positive and Gram-negative bacteria species. The highest zone of inhibitions was observed at the maximum concentration of 500 mg/mL, reaching 23.8 ± 0.79 mm against *E. coli* and 24.5 ± 0.58 mm against *Salmonella typhi* (*S. typhi*). Medium concentration (250 mg/mL), on the other hand, demonstrated comparable efficiency, while lower concentrations (100 mg/mL) showed the least bactericidal activity. Moreover, AgNPs synergistically enhanced the effectiveness of conventional antibiotics, depending on the tested bacterial strain and the antibiotics used. Strongest synergy was observed against *Enterococcus faecalis* (*E. faecalis*), achieving up to 9.84-fold activity with Colistin. Researchers also demonstrated a notable 5.11-fold increase against *Acinetobacter baumannii* (*A. baumannii*) when AgNPs were combined with Ceftazidime [89].

Similarly, Ali et al. examined the antibacterial efficiency of AgNPs green synthesized from *Aspergillus parasiticus* against Methicillin-resistant *S. Aureus* (MRSA). Resulting NPs successfully inhibited the proliferation of MRSA with a MIC value of 25 μg/mL and prevented the biofilm formation caused by MRSA with a clearance rate of 80%. Also, AgNPs promoted the wound healing process in a MRSA-induced skin infection model, leading to complete inhibition of bacterial colonies following 6 days of application [90].

Despite the advantageous aspects of fungi-mediated AgNPs synthesis, many limitations still exist. Lack of standardized synthesis protocols is one of these major drawbacks, as different fungal extracts can produce NPs with different size, morphology and stability. These variations are also influenced by pH, reaction time, AgNO_3_ concentration, and temperature, hindering the reproducibility of the synthesis procedure [67]. Moreover, the mechanisms involved in fungal-based NP production remain largely unknown. While fungi are known to reduce Ag^+^ to Ag^0^, the specific biochemical pathways responsible for NP formation still are not well understood [91]. Another limitation is the relatively slow reaction time compared to conventional physical and chemical methods, which can disrupt large-scale production efficiency. The labor-intensive downstream processing steps required to purify the NPs from fungal residues, including proteins, enzymes, and secondary metabolites, appears as another challenge that can affect the quality of the resulting NPs [5]. Similarly to other microorganism-based methods, fungi-based synthesis also has a risk of contamination which may interfere with the purity of NPs, requiring an aseptic and sterile environment throughout the synthesis process. Considering these, it is important to optimize synthesis conditions and develop standardized protocols to advance fungi-based green synthesis as a promising alternative to conventional synthesis methods.

Although Table 2 provides a summary of the most notable distinctions between bacterial and fungal synthesis, it is essential to give due consideration to the fact that repeatability and scalability continue to be significant obstacles in the field of microbial synthesis. The size, shape, and stability of NPs can vary from batch to batch due to differences in the microbial strains, growing conditions, and metabolic activities that are present. In addition, the process of scaling up from the laboratory to the industrial scale brings up new complications, such as the transfer of oxygen, the regulation of pH, and the uniformity of nutrients in bioreactors.

Furthermore, these difficulties are further complicated by the methodological caliber of many quoted studies. Incomplete physicochemical characterization of the synthesized NPs, inadequate experimental duplicates, or small sample quantities are used in a number of papers. To verify NP production and stability, some studies employ a wide range of analytical techniques (TEM, SEM, XRD, DLS, FTIR, UV-Vis), while others rely on just one or two, which makes cross-study comparisons and reproducibility evaluations challenging.

To overcome these challenges, it is of the utmost importance to establish standardized protocols, implement stringent process control, and implement statistical optimization algorithms, all while simultaneously supporting systematic and standardized reporting of methodological details. Through the completion of these steps, microbial synthesis processes will become more dependable, reproducible, and scalable.

## 5. Activity and Application of Microbial-Based Synthesized AgNPs

Green synthesis of AgNPs from bacteria has emerged as an environmentally friendly and efficient alternative for producing highly reactive and stable NPs. Considering the wide variety of bacterial strains available, the physicochemical properties of AgNPs can be tuned specific to the broad range of applications. This flexibility makes bacteria-based AgNPs an ideal candidate for developing sustainable and innovative solutions across various sectors, including biomedical and environmental applications (Table 3).

Similarly, fungal-based green synthesis of AgNPs offers unique advantages, including controllable and reliable synthesis procedures, along with high yields with an abundance of extracellular enzymes involved in reduction. Given their broad-spectrum antimicrobial properties, fungal-based AgNPs are being explored across various fields as well (Table 4).

### 5.1. Antimicrobial Activity

**Figure 3:**
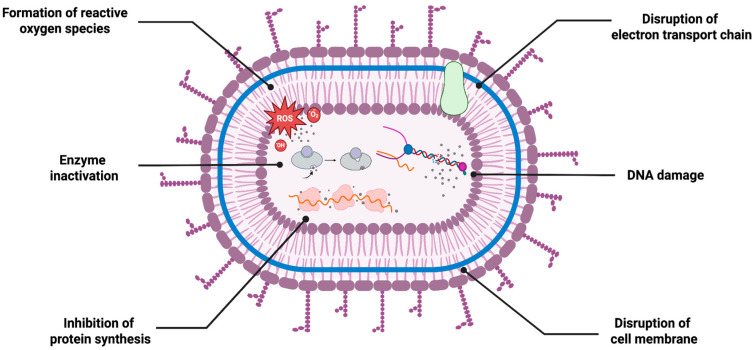
Mechanism of the Antibacterial Action of Green-Synthesized AgNPs [113].

The antimicrobial activity of AgNPs is one of their most studied and discussed properties. These NPs exhibit potent antimicrobial activity against a variety of pathogenic fungal and bacterial species. Thanks to their simultaneous mechanisms of action, they are known as attractive agents in wide-ranging applications including biomedical and environmental applications (Figure 3). Regarding their multi-targeted activity, they are powerful alternatives for reducing the possibility of resistance development and enhancing therapeutic outcomes through synergistic activity potential with other therapeutic agents, including antibiotics. Similarly to their chemically synthesized counterparts, both fungi-based and bacteria-based green-synthesized AgNPs exhibit effective and broad-spectrum of antimicrobial activities, supporting the green synthesis routes in antimicrobial applications.

#### 5.1.1. Antimicrobial Activity of Bacteria-Based AgNPs

Xia et al. green-synthesized AgNPs using biofilm supernatant of *Pseudomonas aeruginosa* PA75, demonstrating their antibacterial, antibiofilm and antitumor activities [114]. The small-sized green-synthesized AgNPs exhibited potent antibacterial activity against several types of MDR bacteria strains, with MIC values ranging from 7.81 to 15.63 μg/mL and minimum bactericidal concentration (MBC) values between 15.63 and 31.25 μg/mL. Notably, gram-negative bacteria were more susceptible to AgNPs than gram-positive strains. Furthermore, AgNPs inhibited biofilm formation inhibition by more than 80% at concentrations between 60 and 120 μg/mL. Additionally, the study also assessed the antitumor activity of the green-synthesized AgNPs.

A similar study synthesized AgNPs using *Bacillus cereus* and determined their biological characteristics including antimicrobial, antioxidant and catalytic properties [83]. Spherical AgNPs with very small particle sizes ranging from 5 to 7.06 nm exhibited significant antimicrobial activity against five MDR strains. The diameters in the ZOI measurements indicated that AgNPs led to an approximate 7–28% increase in the ZOI compared to antibiotics administered alone. Their radical scavenging and catalytic potential were also evaluated with 2,2-diphenyl-1-picrylhydrazyl-hydrate (DPPH) and methyl orange in the industrial wastewater, respectively.

Algburi et al. green synthesized AgNPs using *Lactobacillus acidophilus* (LA-AgNPs) and a mixed *Lactobacillus* culture (ML-AgNPs) to evaluate their antibacterial activity against MDR-bacteria strains [115]. The synthesized NPs exhibited spherical morphology with notable size differences, with an average size of 6.4 ± 1.2 nm for LA-AgNPs and 82.89 ± 12.5 nm for ML-AgNPs. Both types of AgNPs showed strong antibacterial activity against all tested bacteria isolate, producing inhibition zones ranging from 20–30 mm. Interestingly, while combined treatment demonstrated slightly larger inhibition zones ranging from 25–30 mm, ML-AgNPs alone demonstrated higher activity in some cases. MIC analysis confirmed the antibacterial potential of both NP types, with ML-AgNPs showing a broader activity.

One notable application of AgNPs is their use in combination with antibiotics to combat antibiotic-resistant bacteria. Although the exact mechanism is not well established, AgNPs can enhance the efficiency of antibiotics by disturbing cellular membranes, increasing permeability and potentially interfering with bacteria resistance mechanisms [116]. Several studies have reported a synergistic effect, showing enhanced antibacterial activity of both AgNPs and antibiotics in combined treatment.

AgNPs green synthesized from *Pseudomonas aeruginosa* exhibited significant antibacterial activity against several MDR bacteria strains [117]. The synthesized AgNPs demonstrated an average size of 18 nm with aspherical and square morphologies. Well diffusion assay showed that combined treatment of AgNP and antibiotics demonstrated significantly higher inhibition zones than both the antibiotic and NP alone groups. The authors suggested that AgNPs possibly may destroy bacterial cell walls, thus facilitating penetration of antibiotics to the bacteria. An alternative mechanism involving ROS-mediated destruction of membrane lipids and peptidoglycans was also proposed.

Similarly, synergistic effects of green-synthesized AgNPs from *A. baumannii* were evaluated in combination with several antibiotics [118]. The antibacterial efficiency of the AgNPs was tested against multiple strains, including pan drug-resistant, extensively drug-resistant, and MDR bacteria. The AgNPs exhibited MIC values ranging from 8 to 64 μg/mL and MBC values from 16 to 128 μg/mL. Synergistic studies revealed that co-administration of AgNP with antibiotics led to either synergistic or partially synergistic effects, with MIC values of AgNPs reduced by up to four times. Notably, the most significant enhancement was observed for ceftriaxone against *A. baumannii*, where the MIC was dramatically reduced from 1024 to 4 μg/mL. The authors suggested that dual treatment of AgNPs and antibiotics may positively influence the binding affinity towards their targets, thereby improving their penetration and overall efficiency against bacterial resistance. They further emphasized that use of two distinct antibacterial agents would interfere with the bacteria resistant mechanisms, making it more difficult for bacteria to simultaneously develop resistance to both. It was also noted that this combined approach could reduce the concentrations required for effective treatments.

AgNPs green synthesized from *Anabaena variabilis* exhibited significant antimicrobial activity, including synergistic effects when combined with antibiotics and antifungal agents [119]. The spherical, small-sized AgNPs demonstrated potent antibacterial and antifungal activity against a broad range of strains. The synergistic interaction was assessed using a checkerboard assay, where combinations of AgNPs with antibiotics and fungicides were tested. The results revealed fractional inhibitory index values below 0.5 for all combinations, indicating strong synergistic effects between AgNPs and both classes of antimicrobial agents.

These findings collectively demonstrate that bacteria-based green synthesis of AgNPs holds great potential for use in synergistic treatments with antibiotics against drug-resistant pathogens. While conventionally synthesized AgNPs have already shown the ability to enhance efficiency of antibiotics, utilizing green-synthesized nanomaterials offers a more sustainable and biocompatible alternative. This approach could contribute to the development of innovative strategies in antibiotic research.

**Figure 4:**
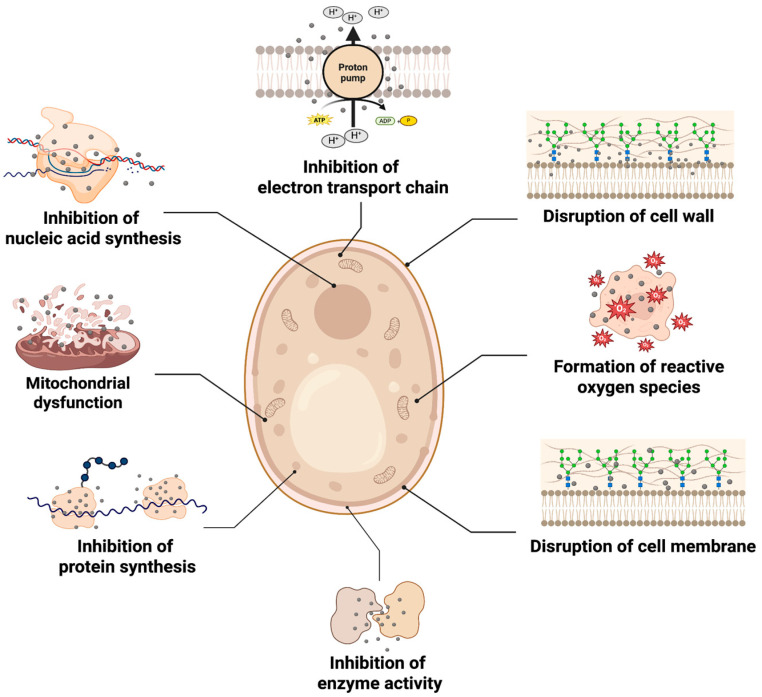
Mechanism of the Antifungal Action of Green-Synthesized AgNPs [38].

Bacteria-based synthesized AgNPs have also demonstrated strong antifungal activity against a wide range of pathogenic fungal strains. Similarly to their role in antibacterial applications, they are also considered promising alternatives to conventional antifungal agents, particularly in addressing antifungal resistance. Given their simultaneous mechanism, along with their small size and high surface reactivity, bacteria-based AgNPs possess high potential for future clinical and antifungal applications (Figure 4).

Elbahnasawy et al. demonstrated the anticandidal activity of AgNPs synthesized from *Rothia endophytica* [92]. Cubic-shaped AgNPs, with an average size ranging from 47 nm to 72 nm, exhibited potent antifungal activity against *Candida albicans* (*C. albicans*), demonstrating notable MIC and MBC values during the experiment. Furthermore, exposure to AgNPs resulted in significant sugar leakage from cultured *C. albicans*, with the extracellular sugar concentration reaching 783.1 μg/mg after 36 h of incubation, two times higher than the control group. Similarly, AgNPs exposure leads to up to four times higher protein leakage compared to control groups at the highest concentration. Finally, signs of lipid peroxidation, indicated by an increased malondialdehyde concentration and visible shrinkage and irregularities of the cytoplasmic membrane under transmission electron microscopy (TEM), were also observed following AgNP treatment. Ajaz et al. synthesized AgNPs using native *Bacillus* sp. strain AW1-2 and demonstrated their antifungal activity against *Colletotrichum falcatum* Went [120]. At the highest tested concentration (20 μg/mL), the AgNPs exhibited approximately 80% inhibition in both liquid and solid media. The morphological analysis further revealed significant disruption of the fungal hyphal structure, along with notable damage to the cell wall, mitochondria, ribosome and chromatin material.

AgNPs synthesized using the marine bacteria *Planococcus maritimus* MBP-2 were evaluated for their antifungal activity, along with antibacterial and cytotoxicity assays [121]. The spherical NPs, with an average size of 24.9 nm, demonstrated significant antifungal activity against a variety of fungal strains, including *A. niger*, *Aspergillus flavus* (*A. flavus*), *Penicillium commune* and *Penicillium digitatum*. In the well diffusion model, AgNPs exhibited significantly stronger activity against *Aspergillus* species (up to 14.33 mm) than the *Penicillum* species (up to 8.66 mm). Similarly, AgNPs green-synthesized from *Shigella flexneri* 29508 exhibited potent antioxidant and antifungal activity [122]. The disc diffusion method revealed that green-synthesized AgNPs showed strong antifungal activity, which was proportional to the dose of administration (2.5, 5, 10 and 20 µg) until the maximum dose reached. Against the two fungal strains (*A. niger* and *C. albicans*), AgNPs demonstrated MIC of 10 and 5 μg/mL respectively. Minimum fungicidal concentrations (MFC) were also determined, being 25 and 40 μg/mL, respectively. The authors further highlighted that AgNPs exhibited fungicidal activity against *A. niger* and fungistatic activity against *C. albicans*. They emphasized similar mechanisms behind this antifungal activity, where Ag^+^ release occurs due to the accumulation of AgNPs on the fungal surface, increasing intracellular ROS synthesis and negatively altering structural formation. This disruption was further investigated by measurements of cellular macromolecules, including DNA, proteins and polysaccharides, where leakage was higher for *A. niger* than *C. albicans* up to 6 h. These results suggest that the antifungal efficacy can be influenced by the type of fungal strain, highlighting the importance of target strain when evaluating the potential of bacteria-based green-synthesized AgNPs for antifungal applications.

#### 5.1.2. Antimicrobial Activity of Fungi-Based AgNPs

Similarly to bacteria-based green synthesis, fungal-based green-synthesized AgNPs also exhibit significant potential in antimicrobial applications, including both antifungal and antibacterial activities. These NPs have gained increasing recognition for their unique advantages in NP production, particularly leveraging the antimicrobial properties of AgNPs.

Kthiri et al. synthesized AgNPs from *Saccharomyces cerevisiae* through two different synthesis protocols, with one involving the exposure of fungal cultures to a static magnetic field (SMF), while the other does not. Spherical AgNPs with average diameters between 11–25 nm (control AgNPs) and 2–12 nm (SMF-exposed AgNPs) demonstrated antibacterial activity against both Gram-positive *S. aureus* and Gram-negative *E. coli*. Control AgNPs had higher MIC values, 40 µg/mL against *E. coli* and 20 µg/mL against *S. aureus*, while SMF-exposed AgNP showing greater potency supported by lower MIC values of 25 µg/mL and 15 µg/mL against *E. coli* and *S. aureus*, respectively. This immense inhibitory effect was attributed to the smaller size of SMF-exposed AgNPs, facilitating more efficient interaction with bacterial cell walls. Further, both types of AgNPs had dose-dependent antibiofilm activity as SMF-exposed AgNPs outperformed their counterparts across all tested concentrations ranging from 25 µg to 100 µg [123].

In another study, Ilahi et al. utilized cellular extracts of endophytic *F. oxysporum* strain NFW16 for the green synthesis of AgNPs. Spherical AgNPs having an average size between 30 to 36.1 nm had MIC of 100 µg against MRSA. AgNPs further showed synergistic effects with antibiotics, such as ciprofloxacin and vancomycin, leading to increased inhibition against *P. aeruginosa* (50%), MRSA (25%) and pus isolated *E. coli* (50%) indicated by higher zone of inhibition values compared to AgNPs or antibiotics alone. Additionally, researchers investigated the antibacterial effectivity of AgNPs adsorbed onto textile products, including cotton and bandage. Maximum ZOI was observed against *S. aureus* and *E. coli* (44 nm) by cotton-adsorbed AgNPs, followed by *Listeria monocytogenes* (43 mm), and smallest zone observed against *Klebsiella pneumoniae* (*K. pneumoniae*) (22 nm). Bandage adsorbed AgNPs had highest ZOI against *E. coli* (42 mm), while the least activity was observed against *K. pneumonia* and *P. aeruginosa* (20 mm) [124].

Recently, Abdel-Kareem et al. synthesized AgNPs from extracellular extracts of *Aspergillus templicola* OR480102 fungi for the first time. Spherical AgNPs with an average size of 17.79 ± 1.36 nm inhibited the proliferation of both Gram-positive and Gram-negative bacterial pathogens. Specifically, AgNPs had MIC values of 2.5, 5, 10, and 20 µg/mL for *S. typhimurium*, *B. subtilis*, *S. aureus* and *E. coli*, respectively. Conversely, fungal extract or AgNO_3_ alone were unable to inhibit proliferation of any tested strains of bacteria. Researchers also revealed the anticancer potential of AgNPs, with an IC_50_ value of 50 µg/mL, following 48 h of treatment against MCF-7 breast cancer cell line [125].

Similarly, Nongthombam et al. utilized extracts of endophytic fungi *Neocosmospora solani* for the green synthesis of AgNPs. NPs with an average size of 362.3 nm and spherical morphology exhibited antimicrobial activity against human pathogens *S. typhi*, *B. subtilis*, *E. coli* and *S. aureus*. In agar well diffusion assay, researchers observed the highest bacterial colony reduction against *S. typhi*, with a ZOI of 16 mm at the highest tested concentration of 60 µL. This was followed by *B. subtilis*, *S. aureus* and *E. coli*, which had ZOI values of 14, 13 and 12, respectively. In general, AgNPs had dose-dependent antibacterial efficiency, with increasing concentrations from 20 to 60 µL leading to larger inhibition zones. They have also highlighted the dose-dependent anticancer potential of the AgNPs against A549 human lung cancer cell line, reducing cell viability to 5% when treated with the highest concentration of 100 µg/mL [126].

Further, Dadayya et al. employed endophytic fungi *Coniothyrium chaingmaiense* for the synthesis of AgNPs (Con-AgNPs) and evaluated their antimicrobial activity. Con-AgNPs was spherical in shape and had an average size of 65.81 nm. Antibacterial assays revealed their dose-dependent efficiency against *E. coli*, *S. aureus*, *P. aeruginosa*, *S. typhi* and *K. pneumoniae* with corresponding ZOI of 14.1 ± 0.17, 13.03 ± 0.05, 14.06 ± 0.11, 13.1 ± 0.17 and 12.06 ± 0.11, at the highest tested concentration of 10 mg/mL. In addition, they revealed the enhanced efficiency of antibiotics when combined with Con-AgNPs, such as Ampicillin and Gentamicin, achieving maximum zone of inhibition when tested against *E. coli*. Similarly, Con-AgNPs also demonstrated dose-dependent antifungal activity against *C. albicans*, *A. brasiliensis* and *A. flavus*. At the highest dose of 10 mg/mL, Con-AgPNPs achieved ZOI values of 12.06 ± 0.11, 12.13 ± 0.03, 13.03 ± 0.05, respectively [127].

In conclusion, fungi-mediated synthesis of AgNPs presents a powerful and versatile strategy for antimicrobial applications. Across various studies, fungal-based AgNPs have consistently demonstrated broad-spectrum antibacterial activities, either alone or in combination with conventional antibiotics.

Similar to their antibacterial activity, fungi-based AgNPs have also demonstrated potent fungicidal effects against a wide range of pathogens [38]. Accordingly. Ahmad et al. synthesized AgNPs from various fungi, *A. flavus*, *Pencillium chrysogenum* and *A. niger*, and investigated their antifungal efficiency. Resulting NPs were between 80 and 100 nm in size, having asymmetrical morphology. Antifungal assays revealed AgNPs’ potent activity towards plant pathogenic fungi, such as *F. oxysporum*, *Rhizopus stolonifer*, *Mucor mucedo*, *Penicillium citrinum* and *Aspergillus terreus*, with ZOI values ranging between 6.66 ± 0.57 and 19.33 ± 0.57 mm [128].

In another study, Ishfaq et al. used *Trichoderma harzianum*, a fungus used to control plant pathogens. AgNPs with an approximate size of 85 nm and both irregular and spherical morphology exhibited inhibitory effects on *Fusarium verticillioides*, one of the main pathogens responsible for maize ear and stalk rots, depending on the incubation time and the concentration used. Longest incubation time of 9 days, compared to 3 and 6, led to highest activity across all tested concentrations of 10, 20, 40, 60 and 80 μg/mL. AgNPs achieved their best fungicidal effect at a concentration of 80 μg/mL, as well as inhibiting sporulation and disrupting cellular integrity [129].

Recently, Oluranti et al. conducted a comparative study on AgNPs green synthesized from three different fungal strains, *Pleurotus ostreatus*, *Agaricus bisporus* and *Agaricus campestris*, which were then evaluated for their activity to inhibit cocoa bean-infecting *A. flavus* (AFD28 and AFD42) and *Aspergillus ochraceus* (AOD40 and AOD45). Resulting AgNPs exhibited varying sizes, 9.22–52.60 nm, 10.24–17.66 nm, and 15.25–45.85 nm, for *A. campestris*, *A. bisporus* and *P. ostreatus*, respectively. AgNPs successfully inhibited the mycelia growth of *A. flavus*, with inhibition rates ranging between 42.31 to 52.73% for AFD28, and 33.83 to 57.07% for AFD42. Concerning the *A. ochraceus*, nps had inhibition rates between 34.64 to 52.36% against AOD40, and 37.43 to 53.56% against AOD45 [130].

Jana et al. synthesized AgNPs from two different strains of endophytic fungi, *Fusarium proliferatum* and *F. oxysporum*, and assessed their antimicrobial activities. Spherical AgNPs with sizes ranging from 3 to 27 nm exhibited dose-dependent fungicidal activity against phytopathogenic fungi *Aspergillus fumigatus*, *A. niger*, *Alternaria alternata*, *Curvularia lunata* and *F. oxysporum*, achieving inhibition rates up to 90% at their highest concentration of 75 μg/mL, compared to 25 and 50 μg/mL. Besides, researchers investigated the effect of AgNPs on fusarium wilt of tomato. A concentration of 150 ppm of AgNPs successfully prevented the infection caused by *F. oxysporum* f.sp. *lycopersici* under greenhouse conditions, highlighting a novel and eco-friendly alternative for the treatment of plant diseases caused by fungi [108].

Overall, both bacterial and fungal-based AgNPs have demonstrated strong and multifunctional antimicrobial activity against bacteria, fungi, and biofilms, often outperforming conventional treatments or enhancing their effects synergistically. While bacteria-based AgNPs offer tunable size and shape control that supports more targeted antimicrobial activity, fungi-based AgNPs are often favored for their ease of large-scale production and higher metabolite output, which can enhance stability and potency. This complementary potential highlights the value of both systems, with bacterial synthesis being advantageous for precision, and fungal synthesis better suited for scalable antimicrobial applications. These trends are also reflected in Table 2, which summarizes the key synthesis parameters, and Table 3, which presents strain-specific applications supporting these observations.

### 5.2. Anticancer Activity

**Figure 5:**
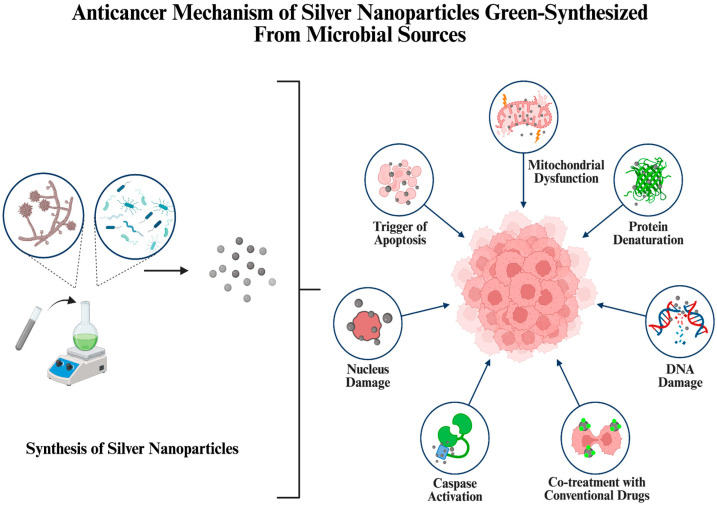
Anticancer Mechanism of Silver Nanoparticles Green-Synthesized from Microbial Sources [131,132].

The biological synthesis of AgNPs offers advantages over physical and chemical approaches, including eco-friendliness, feasibility, and ease of scalability for large-scale production. Silver has served as an antimicrobial agent since antiquity among all new metals. It has garnered considerable interest for its pharmacological, therapeutic, and culinary attributes, with new observations highlighting its substantial efficacy as an anticancer agent (Figure 5) [131,132].

Recent research suggests that AgNPs alter the activity of permeability glycoprotein (Pgp), making treatment more effective against cancer cell lines that have developed resistance. This suggests that these NPs could be a good candidate for combination therapies. In addition to their genotoxic effects, AgNPs cause apoptosis by disrupting DNA strands and causing genomic instability [133,134]. These intriguing mechanistic insights have been the basis for a number of research studies that have focused on the biosynthetic production of AgNPs from microbial sources and their biological applications, particularly in the treatment of cancer. Still, it needs to be highlighted that bacterial and fungal synthesis methods differ in scalability and particle control, which can influence therapeutic performance in anticancer studies, as shown in Table 2.

Pallavi et al. synthesized AgNPs using *Streptomyces hirsutus* strain SNPGA-8 and demonstrated their anticancer activity [132]. The anticancer potential of the AgNPs was evaluated with their significant inhibition of proliferation in human lung carcinoma (A549) cell lines. A dose-dependent response was observed, including notable morphological changes such as cell shrinkage and rounding. At the highest tested concentration (100 μg/mL), AgNPs reduced cell viability to 16%, with an IC_50_ value of 31.41 μg/mL. Additionally, a notable increase in intracellular ROS generation was detected. The AgNPs also exhibited strong antimicrobial activity against various fungal and bacterial species.

In another research, using the endophytic bacterium *Actinobacterial* strain SF23, Pathania et al. synthesized AgNPs and assessed their biological activity. According to the study, AgNPs exhibited dose-dependent cytotoxicity against MCF-7 breast cancer cells and RAW 264.7 macrophages, with MCF-7 exhibiting greater cytotoxicity. LDH leakage increased while cell viability declined. When stimulated with AgNPs, MCF-7 cells generated more ROS than RAW 264.7 macrophages (IC_50_ values of 16.3 and 12.0 μg ml^−1^, respectively). The study comes to the conclusion that AgNPs produced from endophytes have anticancer potential, underscoring their potential for use in future therapeutic applications [135].

Due to the fact that they are affordable, produce a large amount of biomass, produce little toxicity, and require little energy, fungi are commonly used among many different biological agents. The method of producing NPs from fungi is referred to as myco-science. Fungal organisms are responsible for the production of a large quantity of extracellular proteins, enzymes, and metabolites. These biomolecules not only fulfil the role of reducing agents, but they also play a role in NP capping, which means that they have an effect on the size and stability of NPs [136]. Out of the many different fungal genera that are used for the production of NPs, the genus *Fusarium* has been selected by a significant number of researchers. There are a number of advantages associated with the utilization of Fusarium species for the manufacture of NPs. These advantages include quick growth, ease of growing, bulk extracellular production, scalability, reduced costs for handling biomass, safety, and straightforward processing [64].

*Fusarium nygamai* isolate AJTYC1 was used to green-synthesize AgNPs, and El-Ansary et al. thoroughly characterized the resultant NPs using a range of analytical methods, including UV-Vis spectroscopy, TEM, XRD, zeta potential analysis, EDX, and FT-IR. The produced AgNPs had a definite SPR peak at 310 nm and were spherical, with diameters varying from 27.3 to 53.1 nm. Numerous functional groups, including alkanes, alkynes, cyclic alkenes, esters, phenolics, and aromatic amines, were verified by FT-IR spectra. A concentration-dependent decrease in cell viability was seen when their anticancer potential was validated against a number of cancer cell lines, such as HepG2 (hepatocellular carcinoma), HCT116 (colorectal carcinoma), and MCF-7 (breast cancer). In connection with other properties, they showed better antioxidant activity. Along with significant antifungal activity, they also demonstrated potent antibacterial activities against both Gram-positive and Gram-negative bacteria [137]. After screening fungal strains recovered from uncharted soils in the Yellapura region, *Penicillium brasilianum* NP5 was shown to be a strong contender based on morphological, microscopic, and molecular analysis in a different recent study. An alteration in color and an SPR peak at 420 nm indicated that this isolate was used to green-synthesize AgNPs by Rudrappa et al. [136]. With an IC_50_ of 41.93 µg/mL, the AgNPs demonstrated dose-dependent anticancer activity against the MDA-MB-231 breast cancer cell line and demonstrated potent antimicrobial activities against certain human infections. Their anticancer potential was further confirmed by apoptosis studies based on flow cytometry.

The severe side effects and limited solubility of the widely used chemotherapeutic drug paclitaxel (PTX) pose substantial restrictions. In order to address these issues, new research has focused on creating novel nanocomposites. One method had the endophytic fungus *Aspergillus fumigatiaffinis* produce AgNPs, which were then conjugated with PTX. This nanoconjugate was found to be 5 to 10 times more effective than AgNPs alone in suppressing MCF-7 breast cancer cells in in vitro tests, with an IC_50_ value of 1.7 µg/mL. Additional confirmation of its capacity to trigger cell death was provided by apoptosis experiments. An attractive technique for advanced cancer treatment, this green nanoplatform shows how chemotherapeutic drugs can be combined with AgNPs to improve tumor-targeted efficacy and decrease systemic toxicity [138].

One in 53 Indian women have cervical cancer, which is treated mostly with chemotherapy and radiation. However, side effects continue, requiring side effect-free or low-cost drugs. Recent research aimed to synthesize AgNPs utilising metal-tolerant pre-isolated *A. niger* and evaluate the anticancer efficacy of these synthesized AgNPs against the HeLa cell line via an in vitro cytotoxicity assay. Lan Chi et al. selected metal-tolerant fungi species to assess their capacity to decrease, encapsulate, and stabilize AgNPs. The AgNPs were assessed for their anticancer effectiveness against HeLa cell lines and shown significant cytotoxicity. At a concentration of 100 g/mL, the biofabricated AgNPs demonstrated 70.2% inhibition, with an IC_50_ value of 66.32 g/mL [139]. Similarly to this, Akther et al. synthesized AgNPs using an endophytic fungus that was separated from the therapeutic herb *Catharanthus roseus* (Linn.). ITS sequencing revealed that the endophytic fungus was *Botryosphaeria rhodina*. AgNPs’ in vitro anticancer activity was evaluated using A-549 cells. Under in vitro circumstances, the produced AgNPs showed efficacy in scavenging free radicals and causing nuclear and DNA fragmentation, two apoptotic hallmarks, in lung (A549) cancer cell lines. The findings showed that the synergistic cytotoxic action against cancer cells might be due to the natural biomolecules in the endophytic fungi that were integrated into the NPs. The cytotoxicity IC_50_ of AgNPs against A549 cells was shown to be 40 μg/mL [140].

In a recent study, Bishoyi et al. used an environmentally friendly and green method to green-synthesize AgNPs utilizing the cyanobacterium *Oscillatoria salina* (Os-AgNPs). In vitro, the Os-AgNPs also showed antiproliferative action against cancer cell lines, such as MDA-MB-231 (breast cancer) and HeLa (cervical cancer). Additionally, Swiss mouse models were used for in vivo toxicity evaluations in order to identify possible harmful effects. The green-synthesized Os-AgNPs demonstrated potent antibacterial activity in addition to their antiproliferative characteristics. With inhibition zones ranging from 15 to 20 mm, antibacterial tests demonstrated significant inhibition against both Gram-negative strains of bacteria, such as *E. coli*, *K. pneumoniae*, and *P. aeruginosa*, and MDR Gram-positive strains, such as *S. aureus*, *Streptococcus pyogenes*, and *E. faecalis*. Furthermore, the NPs produced inhibition zones of 20–30 mm and showed significant antifungal activity against *Trichophyton rubrum* and *Candida tropicalis* [141].

In conclusion, because of their capacity to trigger apoptosis, produce ROS, and stop the growth of tumor cells, green-synthesized AgNPs originating from a broad range of microbial and fungal sources hold tremendous potential in anticancer treatment. When combined with chemotherapeutic drugs, these NPs not only increase drug efficacy but also offer a scalable, affordable, and environmentally friendly substitute for traditional therapies. Their versatility is further demonstrated by their biocompatibility and broad-spectrum antibacterial action. While bacterial-based AgNPs provide more uniform and tunable physicochemical properties that support consistent therapeutic effects, fungal-based AgNPs may offer enhanced potency due to the additional bioactive metabolites produced during synthesis. This distinction highlights their complementary strengths: bacterial synthesis favors precision and reproducibility, whereas fungal synthesis provides scalability and potentially stronger biological activity. Translating these nanomaterials into innovative therapeutic platforms will require ongoing research into their mechanisms, safety profiles, and clinical application.

### 5.3. Agricultural Applications

**Figure 6:**
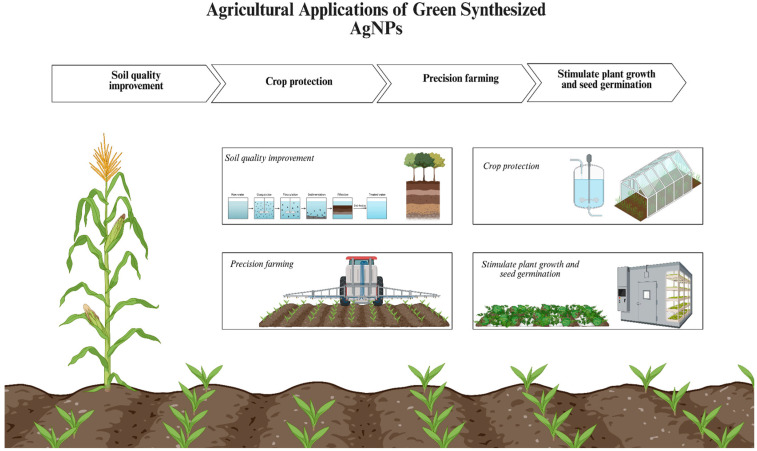
The General Summary of Agricultural Applications of Green Synthesized AgNPs [143,145].

One of the most important industries that directly or indirectly supports people with food is agriculture. Despite the consistent supply of natural resources from farming, the demand for all of the aforementioned is being driven up by the ever-increasing population [142]. This means it is crucial to focus on researching agricultural expertise and using new technology to produce crops efficiently [143]. Seed germination, agricultural disease detection, soil quality improvement, nano-fertilizers, and other related topics continue to draw the attention of researchers. The multidisciplinary study of nanotechnology is providing a foundation for addressing this intriguing industry. Numerous biotic and abiotic stressors are constantly affecting agricultural systems. The primary biotic restrictions influencing crop productivity on agricultural fields are plant diseases, which could result in a global food catastrophe. Numerous studies have demonstrated the significant effect that employable NPs, including AgNPs, can play in controlling plant diseases and fostering plant development (Figure 6) [144]. Better control and conservation of inputs for plant and animal production are two ways in which applications helped by nanotechnology could revolutionize agricultural production [143]. AgNPs have attracted a lot of interest among these NPs because of their broad-spectrum antibacterial capabilities and comparatively low toxicity to plants [145].

According to a number of studies, AgNPs can inhibit a wide range of bacterial phytopathogens, including *Pseudomonas syringae* pv. tomato DC 3000 [146], *Acidovorax oryzae* strain RS-2 [147], and *Clavibacter michiganensis* subsp. *michiganensis* [148]. Recent research adds to this evidence by demonstrating how well green-synthesized AgNPs work to control ailments in rice, one of the most important staple crops in the world. Balamurugan et al. demonstrated the antimicrobial activity of AgNPs synthesized using *Pseudomonas fluorescens* against brown leaf spot disease in rice [149]. The particles, with an average particle size of 50 nm, were tested for their fungicidal potential against *Cochliobolus miyabeanus* (*C. miyabeanus*). Upon exposure to the AgNPs, *C. miyabeanus* exhibited notable morphological alterations, including swelling and bursting of hyphal tips and potential disruption on cell wall synthesis, and the induction of osmotic stress.

*Bacillus megaterium*, *Bacillus cereus*, *Bacillus amyloliquefaciens*, *Bacillus flexus*, and *Bacillus subtilis* are among the bacteria of the genus Bacillus that are used to synthesize green AgNPs. These compounds have been extensively researched and are sustainable [150,151]. Ibrahim et al. used the endophytic bacterium *Bacillus siamensis* strain C1, which was obtained from the medicinal plant *Coriandrum sativum*, to carry out an environmentally benign process for the production of AgNPs. The synthesized AgNPs were confirmed to have a spherical form and a size range of 25–50 nm by means of a variety of techniques, including UV-Vis spectroscopy, FTIR, XRD, SEM, TEM, and EDS. The new green-synthesized AgNPs showed promise in promoting plant growth and shielding rice plants from bacterial leaf blight and bacterial brown stripe infections, according to the study’s findings overall [144].

To counteract bacterial leaf blight (BLB), which is caused by *Xanthomonas oryzae* pv. *oryzae* (*Xoo*) and is one of the most destructive biotic stresses impacting rice farming, an alternate approach was applied. AgNPs were used as a possible antibacterial agent to get around the drawbacks of chemical disease control methods. A natural strain of Bacillus cereus SZT1 was isolated and taxonomically described using 16S rRNA gene sequencing, and these AgNPs were made from it utilizing a green method. In order to ascertain the green-synthesized AgNPs’ potential for sustainable rice disease management, their effectiveness against *Xoo* was then assessed. For *Xoo*, AgNPs showed strong antibacterial action (24.21 ± 1.01 mm). By dramatically raising plant biomass, lowering cellular ROS concentrations, and raising antioxidant enzyme activity, AgNPs were shown to be efficient BLB fighters for this disease [152].

According to a recent study, endophytic fungus linked to medicinal plants are becoming more well-known for their potential uses in green nanotechnology. By bioreducing Ag^+^, the endophytic bacterium SQ3, which was isolated from Panax notoginseng and identified as *Chaetomium globosum*, showed that it could green-synthesize AgNPs. These AgNPs showed strong antifungal properties against *Helminthosporium maydis*, *Botrytis cinerea*, and *Phytophthora infestans* in in vitro tests. Additionally, antibacterial activity was verified; inhibition zones of 17.5 mm, 15.2 mm, and 13.4 mm were noted against *Bacillus subtilis*, *Escherichia coli*, and *Staphylococcus aureus*, respectively. Additionally, cherry tomatoes treated with AgNPs after harvest showed a significant decrease in Botrytis cinerea infection, a delay in quality degradation, a reduction in weight loss, and preservation of fruit firmness and nutritional indices such as soluble solids and titratable acidity [153].

To sum up, AgNPs green-synthesized by environmentally benign methods, especially with the help of microbial sources including Bacillus species and endophytic fungi, have demonstrated tremendous promise as multipurpose agents in contemporary agriculture. They are ideal options for sustainable crop production and disease control because of their broad-spectrum antibacterial activity, effects that promote plant development, and postharvest protective qualities. While bacterial-based AgNPs allow for precise control over NP characteristics and can be tailored for targeted crop protection, fungal-based AgNPs provide advantages in scalability and cost-effectiveness, making them more suitable for large-scale agricultural applications. This complementary potential enables strategic selection of synthesis methods depending on specific agricultural needs. This observation is further supported by the strain-specific examples listed in Table 3, many of which are linked to crop protection and growth promotion. To further integrate them into feasible agricultural systems, further research is needed to understand their modes of action, field-level effectiveness, and environmental safety.

### 5.4. Environmental Applications

Another field in which AgNPs have gained significant attention is the environmental and industrial sectors. Due to their unique physicochemical properties, such as high stability, large surface area, and antimicrobial activity, AgNPs have been extensively researched for applications in biosensing, imaging, wastewater treatment and catalysis. In this context, eco-friendly nanomaterials, including green-synthesized AgNPs, are emerging as green-based alternatives, offering distinct advantages for industrial applications [154]. Both fungal and bacteria synthesis routes provide a preferable approach for AgNP production, yielding highly stable, biocompatible and reactive particles that enhance environmental remediation and industrial processes.

The catalytic activity of AgNPs is connected with their large surface area and SPR properties. AgNPs facilitate electron transfer between donor and acceptor molecules, accelerating redox reactions such as the degradation of organic dyes and pollutants [155]. Their surface atoms provide active sites that enhance reactant adsorption and lower activation energy. Thus, the presence of functional groups can facilitate electron transfer and enable reactant adsorption. Surface molecules, such as proteins, enzymes, or polysaccharides derived from biological synthesis routes, can create active sites that contribute to improved catalytic efficiency in processes like dye degradation, pollutant reduction, and other redox reactions. These effects have been widely associated with AgNP-based catalysts, where surface composition and structure strongly influence catalytic behavior [156].

Mechouche et al. synthesized AgNPs from *Streptomyces tuirus* strain and evaluated their photocatalytic dye degradation ability on methylene blue [157]. The photocatalytic degradation was tested under both UV light and sunlight irradiation (for 6 h between 10 a.m. and 4 p.m.), with and without presence of AgNPs. After the addition of AgNPs, the stages of degradation were confirmed by the observed absorption peak at 664 nm. However, there was a slight reduction in the absorption peak under UV light irradiation, while sunlight irradiation allowed for continuous dye degradation in presence of the metal catalyst. Additionally, dye degradation without AgNPs was found to be insignificant. A significant difference in degradation rates was observed depending on the light source. Sunlight irradiation resulted in an effective 71.3% degradation of methylene blue, whereas UV irradiation only achieved 11.25% degradation. The authors also proposed a degradation mechanism of methyl blue, where SPR characteristics of AgNPs allows excitation of surface electrons, leading to ROS-mediated degradation of the dye.

Similarly, AgNPs synthesized from *Cytobacillus firmus* were evaluated for their ability to degrade methylene blue and their effectiveness against the phytotoxicity of dye solutions [158]. Sunlight irradiation of methylene blue with AgNPs was conducted under varying AgNP concentrations (0.25 to 1.0 mg/mL) and contact times (2 to 12 h). The results revealed that biodegradation activity of AgNPs exhibited dose-dependent efficiency, with higher activity observed under sunlight compared to dark conditions. At a concentration of 0.25 mg/mL over 12 h, the decolorization ratio was 19.8 ± 0.02%, whereas it increased significantly to 98 ± 0.065% at a concentration of 1 mg/mL after 8 h. Furthermore, the authors conducted a phytotoxicity test to evaluate the environmental impact of dye degradation. Using broad beans (*Vicia faba*), the crops were treated with methylene blue with and without AgNPs (1 mg/mL, 8 h). The results showed that methyl blue treatment without AgNPs inhibited both shoot and root growth, while AgNP treatment significantly improved seed germination.

On the same basis, Mustafa et al. conducted a comparative study on the dye removal efficiency of AgNPs which were green synthesized from three different sources of fungi, *Fusarium begonia* (Fu-AgNPs), *Mucor* spp. (Mu-AgNPs), and *Cladosporium* spp. (Cl-AgNPs). AgNPs had spherical morphology and varying sizes, 4.8 ± 1.7 nm, 9.96 ± 3.81 and 12.75 ± 4.7 nm, for Mu-AgNPs, Fu-AgNPs and Cl-AgNPs, respectively. Fu-AgNPs had the highest removal rates against various dyes, such as Acid blue 62 (AB62), Reactive (RR), Basic Yellow (by) and Everzol Black B (EBB), achieving a minimum of 89.5% and up to 98.3% following 1 h of reaction time. However, researchers stated that for the further optimization of the dye degradation processes, integration of advanced characterization techniques are required to have a comprehensive understanding of physicochemical properties of the dyes [159].

In a recent study, Saied et al. synthesized AgNPs from *Aspergillus hiratsukae* and assessed their dye-degradation efficiency. Spherical AgNPs, ranging between 16 to 31 nm, had strong photocatalytic activity against acid black 2 (nigrosine) under two separate experimental conditions. Following 300 min of exposure to AgNPs at a concentration of 2.0 mg/mL under the light, nigrosine dye was degraded by 93.3% ± 0.95%. However, in the absence of light, AgNPs reached 51.8% ± 0.83% of dye removal at the same tested concentration and incubation time [160].

Sutar et al. utilized a novel fungal strain, *Beauveria* sp. MTCC 5184, for the green synthesis of AgNPs and evaluated their dye degradation activity against two textile dyes (Reactive Blue HERD and Orange M2R cold brand) having a concentration range between 60 and 100 ppm. AgNPs, with an average particle size of 58 nm and variable morphology, achieved their highest efficiency with degradation rates between 96% to 99% against Blue HERD and Orange M2R at a concentration of 80 ppm. In contrast, when the dye concentration was increased to 90 and 100 ppm, a decrease in AgNPs’ activity was observed. The researchers also investigated the effectiveness of AgNPs against a mixture of dyes at a concentration of 80 ppm. Within 35 min, both dyes were effectively degraded, with the blue dye being decolorized at a faster rate in comparison to the orange dye [161].

Abd Elghaffar et al. synthesized AgNPs from *Aspergillus luchuensis*. Spherical AgNPs with sizes ranging between 16 to 18 nm displayed high catalytic efficiency against safranin dye under light irradiation, achieving a maximum decolorization percentage of 100% following 6 h of exposure [162].

From another perspective, Basheer et al. presented an alternative approach to prevent environmental contaminants, especially focusing on water pollutants. In their study, they have synthesized AgNPs from marine fungi, *Aspergillus terreus*, *Aspergillus oryzae*, *Penicillium simplicissimum* and *Aspergillus terreus*, to assess their capability of inhibiting the proliferation of pathogenic microorganisms. Spherical AgNPs with a size range of 3.8 to 23 nm, exhibited antimicrobial activity against 13 different pathogens, including 8 bacteria, 3 fungi and 2 yeasts. The highest antibacterial activity observed by *A. japonicus* and highest antifungal activity by *P. simplicissimum*, at corresponding concentrations of 5 mM and 8 mM. These results demonstrate the potential of AgNPs synthesized from marine fungi for application in the treatment and bioremediation of contaminated water bodies to improve the overall water quality [163].

Similarly, Namasivayam et al. assessed the wastewater treatment effectiveness of AgNPs green synthesized from a fungal consortium including a combination of four different fungal strains, *Metarhizium anisopliae*, *Penicillium rubrum*, *Beauveria bassiana* and *Nomuraea rileyi*. The effect of NPs on wastewater treatment was focused on the analysis of various parameters, including the viable count of waterborne pathogenic bacteria, biofilm inhibition, chemical oxygen demand (COD), dissolved oxygen, total dissolved solids (TDS) and biological oxygen demand (BOD). All the parameters had a decrease in both tested incubation periods of 10 and 30 days, with maximum reductions observed at 30 days of treatment. Specifically, AgNPs reduced the total viable count of *E. coli* to 25 × 10^3^ CFU/mL and *E. faecalis* to 10 × 10^3^ CFU/mL, compared to control samples having initial concentrations of 67 × 10^5^ and 22 × 10^5^, respectively. Biofilm inhibition was also inhibited by 65%. Researchers further evaluated the effect of chitosan coating on the activity of AgNPs (CS-AgNPs), which showed increased reductions in TDS, COD, BOD, biofilm inhibition, bacterial reduction and alkalinity, outperforming the free AgNPs [164].

In the context of environmental applications, AgNPs derived from both sources have demonstrated high efficiency in water disinfection, dye degradation, and pollutant removal. Bacterial AgNPs provide stable and reactive NPs suitable for targeted remediation processes, whereas fungal AgNPs often achieve higher yields at lower production cost, making them attractive for large-scale environmental treatment. This reflects the versatility of microbial synthesis approaches described in Table 2 and demonstrated by the examples in Table 3. This balance between precision and scalability underscores their complementary roles in sustainable environmental technologies.

## 6. Toxicity & Safety Concerns

Toxicity and safety considerations of AgNPs have increasingly become a critical factor that is considered in their application, regardless of the synthesis method. Although green synthesis using microorganisms such as bacteria and fungi is often promoted as a safer and more sustainable alternative to conventional chemical methods, this approach does not completely eliminate potential biological and environmental risks. Once released into biological systems or the environment, AgNPs can induce a range of adverse effects through mechanisms such as oxidative stress, disruption of membrane integrity, interference with essential biomolecules, and accumulation in tissues [165]. Their physicochemical characteristics, including size, shape, surface charge and capping agents derived from microbial metabolites, play a decisive role in determining their toxicological behavior [166,167].

The increasing use of AgNPs has raised concerns about their release into natural ecosystems, especially aquatic environments, where they can persist and accumulate. Once discharged through industrial, domestic, or agricultural routes, AgNPs enter surface waters and sediments, interacting with environmental and biological components [168]. Aquatic organisms such as certain algae and fish species can take up AgNPs through membrane binding or endocytosis. Inside cells, they may dissolve and release Ag^+^, leading to bioaccumulation and potential food chain transfer. However, the long-term effects of low-level exposure and detoxification mechanisms remain poorly understood, highlighting the need for standardized ecotoxicological evaluations. Although green synthesis is often considered safer and more sustainable than conventional methods, recent evaluations suggest that its environmental impact can vary depending on several factors, such as process scale and resource use [169].

Recent work by Castro et al. worked on the toxicity threshold values for AgNPs in aquatic and sediment compartments for development of environmental safety policies [170]. Using 82.8 nm AgNPs and data from multiple taxa, algae, microcrustaceans, fish embryos, and nematodes, they estimated predicted no-effect concentrations in the range of 0.07 to 0.35 µg L^−1^. They also discussed that management measures or regulatory limits will be needed as AgNP use expands. These threshold values can guide regulators in setting safe exposure limits and highlight the need for clear testing standards and safety measures for green-synthesized AgNPs.

Therefore, evaluating the cytotoxicity, ecotoxicity, and overall safety profile of green-synthesized AgNPs is essential for ensuring their responsible and sustainable application in biomedical, agricultural, and environmental fields. Several representative studies are highlighted to provide a more comprehensive overview of the biological effects of AgNPs under different exposure conditions.

In a recent study, Khan et al. comprehensively assessed the toxicological effects of chemically synthesized AgNPs on *Oncorhynchus mykiss*, also known as rainbow trout. Fish were exposed to AgNP concentrations of varying concentrations (0.2, 0.8, and 1.4 mg/L) for 21 days to assess physiological, biochemical, histopathological and behavioral alterations. At higher concentrations AgNPs led to notable reductions in hemoglobin, hematocrit and red blood cell counts, along with increases in white blood cells indicating immune activation. Biochemical assays revealed dose-dependent decreases in total protein and albumin, along with increases in triglyceride and cholesterol levels, with the 1.4 mg/L group being most affected. Similarly, administration of the higher doses of AgNPs induced oxidative stress, evidenced by reduced activities of antioxidant enzymes superoxide dismutase (SOD), catalase (CAT) and glutathione-S-transferase (GST). Histopathological studies indicated hyperplasia, necrosis and lamellar fusion in the gills, as well as significant liver damage, particularly at the highest exposure level of 1.4 mg/L. Researchers also observed reduced feeding and erratic swimming through behavioral assays on fish exposed to higher AgNP concentrations [171].

From another perspective, Kim et al. investigated the embryotoxic effects of AgNPs utilizing a rat whole embryo culture model. Testing AgNPs at different concentrations, 1.67, 5, and 15 μg/mL, the researchers examined AgNPs’ impact on embryogenic growth and development following 48 h of exposure. In particular, 15 μg/mL of AgNPs led to retardation in embryonic growth and differentiation, together with an increased occurrence of morphological abnormalities such as abnormal axial rotation, open neural tube, absent optic vesicle and growth retarded. Concerning the 5 μg/mL AgNP group, results also revealed decreases in the embryonic otic system, somite number, and total morphological score. On the other hand, no adverse effects were associated with AgNPs at a concentration of 1.67 μg/mL in terms embryoning development and growth [172].

Even though the green-synthesized AgNPs are regarded as safer alternatives to their chemically synthesized counterparts, several studies highlight their toxic effects.

Tareq et al. investigated the in vivo toxicity potential of AgNPs synthesized from *Psidium guajava* leaf extract on rats [173]. The histological analysis of brain tissues and TEM imaging revealed the presence of AgNPs in the brain. While lower concentrations (0.5 mg/kg) did not show notable alterations, medium concentrations (5 mg/kg) caused structural changes in neurons and their myelinated membranes. Compared to the control group, the highest concentration of AgNPs (10 mg/kg) led to an approximate 38% increase in oxidative stress, whereas low and medium concentrations did not significantly increase it. Another study tested toxicity of *Elaeocarpus serratus* fruit extract synthesized AgNPs against marine microcrustacean *Artemia nauplii* (*A. nauplii*), along with their biological evaluation [174]. The morphological analysis showed that increasing concentrations of AgNPs (ranging from 3 to 15 mg/mL) accumulated in the gut and mouth regions of *A. nauplii*. The dose-dependent toxicity of the particles was further demonstrated by a reduction in hatching percentage, decreasing from nearly 90% to about 20% at the highest concentration. In addition, the mortality rate increased from approximately 5% to 69%, further confirming the dose-dependent toxic effect. The toxicity potential of green-synthesized AgNPs has also been investigated in zebrafish, focusing on nephrotoxic effects [175], and in environmental settings assessing their ecotoxic impact in water and soil [176].

Together, these findings demonstrate that AgNPs can exert notable toxic effects in a concentration and condition dependent manner, affecting both cellular and organismal systems. Recognizing and addressing these risks through standardized assessment and regulatory measures is essential to ensure the safe and responsible application of green-synthesized AgNPs.

## 7. Conclusions & Future Perspectives

Using bacteria and fungi to create AgNPs has the benefits of making them more sustainable, affordable, and customizable compared to traditional chemical methods, as this paper has explained. Utilizing microorganisms, particularly fungi and bacteria, in NP synthesis enhances stability, biocompatibility, and reactivity by leveraging their enzymatic and metabolic processes. As a result, these green synthesis methods present promising alternatives for the development of improved, eco-friendly, and sustainable technologies. However, while these microbial approaches have proven to be successful in enhancing the stability, biocompatibility, and reactivity of NPs, further optimization is necessary to maximize their potential.

A recent bibliometric study on green synthesized AgNPs emphasizes the requirement of further investigation on synthesis methods and antibacterial mechanisms of AgNP, while also highlighting the importance of optimizing green synthesis processes to improve NP production efficiency and stability [177]. Furthermore, the study identifies key research keywords that represent future research, including anticancer activity, antioxidant properties, in vitro studies and use of leaf extracts. Given the environmentally friendly nature of green-synthesized AgNPs and the reduced usage of toxic substances, fields closely connected to medical studies, such as antioxidants and anticancer research, are expected to be major topics in the near future. Plant extracts are the most widely studied source for green-based NP production, with AgNPs being the most synthesized metal oxide NPs [178]. With the growing trends in research and the increasing focus on plant-based AgNPs, plant extracts are likely to remain a central topic of interest in future studies.

Due to their small size, highly reactive nature, and ability to generate efficient ROS, AgNPs exhibit significant toxicity potential for biological systems. The synthesis method, particularly when chemically based, and the physicochemical properties, such as size, shape and surface charge, significantly contribute to their toxicity [179]. In contrast, green-based synthesis of AgNPs, which utilized biocompatible and stabilized components for reduction, generally reduces the toxicity issues. As a result, AgNPs are more suitable to be included in biomedical applications with enhanced biocompatibility. However, further investigation is still needed to evaluate both short- and long-term toxicity, particularly in terms of large-scale production and widespread application in environmental and health sectors [180].

A recent comprehensive update on AgNP toxicity highlights that AgNPs released into the environment can undergo certain reactions facilitated by natural substances, such as sulfide and chloride, which can significantly influence their toxicity [181]. This environmental toxicity could negatively impact agricultural applications by disrupting the microbial balance in water on soil. However, numerous studies also emphasize the potential benefits of green-synthesized AgNPs in agriculture, including their antimicrobial activity against plant pathogens, their ability to remove heavy metals from wastewater, and their positive effects on plant growth parameters [75,153,182,183,184]. These findings highlight the importance of green synthesis of AgNPs, which not only reduces the risk of negatively impacting the environment through excessive reactive AgNPs but also enhances agricultural applications by utilizing the multifunctional characteristics of AgNPs in a controlled manner.

Looking ahead, in order to fully explore the potential of green synthesis, a number of crucial research gaps must be filled. To clarify the molecular mechanisms and microbiological components involved in NP nucleation, growth, and stabilization, comprehensive mechanistic research is necessary. Furthermore, in order to reliably assess the safety of green-synthesized AgNPs for biomedical and environmental applications, as well as to provide dependable comparability between investigations, standardized toxicity evaluation techniques must be developed. In order to increase synthesis efficiency, particle homogeneity, and repeatability, future research should also focus on process optimization and scalability, including the application of statistical tools such as RSM. Deeper understanding of microbial–NP interactions and the support of logical process control for commercial translation may be possible through the integration of omics-based methodologies and precise analytical techniques.

The optimization of microbial NP synthesis is a highly interesting future direction that can be pursued through the application of genetic engineering and synthetic biology methods. Through the modification of essential metabolic pathways, enzyme expression profiles, and secretion mechanisms, it is feasible to establish a more exact control over the size, shape, yield, and surface functionality of NPs. Recent developments in genome editing using CRISPR and microbial pathway engineering have made it possible to create strong tools that may be used to build microbial strains that are suited to specific needs and have enhanced capabilities in producing NPs. An acceleration in the development of biosynthesis platforms that are scalable and predictable could be achieved by the integration of these approaches with bioprocess optimization.

Both bacterial and fungal synthesis processes have their own unique advantages and disadvantages when it comes to scaling up their operations, but both approaches provide significant advantages from an industrial and economic point of view. In general, bacterial systems are advantageous because they have rapid growth rates, straightforward culture media, and well-established bioreactor technologies, all of which can reduce the amount of time required for production and the expenses associated with it. There is a possibility that additional downstream processing steps, such as the removal of endotoxins and the recovery of intracellular NPs, could enhance the complexity and cost of the process. Fungal systems, on the other hand, often require longer cultivation times and richer media, but they permit the synthesis of extracellular NPs, which simplifies purification and may result in a reduction in costs further down the line. The contrasts between the two methods suggest that bacterial synthesis may be more advantageous for high-value applications that require accuracy, whereas fungal synthesis may be more ideal for manufacturing in large quantities that is more cost-effective, depending on the industry that is being targeted.

In conclusion, although microbial-based synthesis of AgNPs presents an advantageous route over conventional methods, further research is required to fully optimize their potential. Lack of understanding of the microbe-based synthesis mechanisms, optimization of the synthesis protocols, and toxicity concerns are the major challenges that need to be addressed. Overcoming these will facilitate more efficient, sustainable and safer production of AgNPs, ultimately expanding their application in fields such as biomedicine, agriculture and industry.

## Figures and Tables

**Table 1:** Advantages and Limitations of Conventional and Green Synthesis of AgNPs.

Synthesis Method	Advantages	Limitations	References
Chemical	Rapid synthesis Significant control over physicochemical property of NPs	High energy consumption Use of toxic chemicals High heat generation Limited applicability for clinical use	[12,22]
Plant-based	Cost effective High availability of source Eco-friendly and sustainable Effective phytochemical act as reducing and stabilizing agents	Variability and limited availability in certain plant species Limited control over the physicochemical properties	[18,23,24]
Bacteria-based	Convenience in purification Metabolically versatile strains Tunable physicochemical properties with diverse choice of strains and synthesis parameter manipulation	Possibility of contamination Longer synthesis time, especially compared to plant-based approach	[18,25]
Fungi-based	High metal tolerance Capable of generating large amounts of extracellular enzymes Controllable during downstream processing	Possibility of contamination Longer synthesis time, especially compared to plant-based approach	[18,23]
Algae-based	Rich functional biomolecules Producing NPs with diverse physicochemical properties Fast growth and easily scalable Low-cost cultivation Potential for bioremediation	Limited knowledge of synthesis mechanisms High maintenance	[24,26,27]

**Table 2:** Comparison of Bacterial and Fungal Synthesis Routes Across Key Process Parameters [5,18,25,62,63].

Key Process Parameter	Bacterial-Based Synthesis	Fungi-Based Synthesis
Size/shape	pH, temperature, incubation period, metal precursor concentration, and bacterial strain substantially affect nanoparticle size and form. Controlling these factors allows the creation of spherical, triangular, and rod-like forms, but strain variability can cause less uniform distributions without process control.	Fungal systems facilitate more consistent size and shape distributions through the production of extracellular proteins and polysaccharides that function as natural reducing and capping agents. Under mild circumstances, these biomolecules facilitate the production of stable, precisely defined NPs.
Ease of scaling	Scaling up is easy with fast growth, cheap media, and proven bioreactor technology. Removing endotoxins and recovering intracellular products can complicate downstream processing.	Extracellular synthesis simplifies downstream recovery and may reduce costs at larger scales, despite the necessity of longer cultivation times and enriched media.
Stability	Moderate stability is attained via the cell wall and extracellular polymeric compounds; supplementary stabilizers may be necessary.	Enhanced colloidal stability is attributed to secreted proteins that function as natural stabilizers, albeit with potential partial obstruction of catalytic sites.
Localization of process	Predominantly intracellular or periplasmic, with some strains capable of extracellular synthesis.	Primarily extracellular, facilitating easier nanoparticle recovery and purification.
Reproducibility	Even though strain variability may have an effect on the results, it is possible to obtain high repeatability. This can be accomplished under specific conditions and through statistical optimization (for example, RSM).	The secretion patterns change depending on the growth phase and the composition of the medium, which might result in batch-to-batch variability if the process is not well regulated.
Cost	The use of simple media and rapid production cycles results in low operating costs; however, the addition of extra purification stages (such as the removal of endotoxins and the disruption of cells) can result in an increase in the overall cost.	Higher expenses for the medium and cultivation, but reduced costs for downstream processing as a result of extracellular production; typically cost-effective when produced in large quantities.

**Table 3:** Applications and Properties of Bacteria-Based AgNPs.

Synthesized Bacterial Strain	Application Type	NP Property	Results	References
*Streptomyces enissocaesilis* BS1	Antimicrobial Biomedical	Average particle size of 32.2 nm Spherical morphology SPR peak at 434 nm	Antibacterial activity against multiple strains Antibiofilm activity Anticancer activity against MCF-7 and Caco-2 cancer cell lines	[10]
*Rothia endophytica*	Antimicrobial	Particle size between 47 and 72 nm Cubical morphology Zeta potential of −5.06 ± 0.52 mV SPR peak at 410 nm	Antifungal activity against *Candida albicans* ATCC 10231	[92]
*Cytobacillus firmus*	Antimicrobial	Average particle size of 20 nm Spherical morphology SPR peaks at a range of 400–470 nm	Antibacterial activity against *Edwardsiella tarda* Dose-dependent antibiofilm activity against *Edwardsiella tarda*	[93]
*Lactobacillus plantarum*	Biomedical Antimicrobial	Particle size between 40 and 50 nm Spherical morphology Zeta potential of −78.8 mV SPR peak at 436 nm	Antioxidant activity Antibacterial activity against multiple strains	[94]
*Cedecea lapagei*	Antimicrobial	Particle size between 10 and 40 nm Spherical, triangular and hexagonal Zeta potential of −15.3 mV SPR peaks at a range of 400–500 nm	Antibacterial activity against multiple strains Antibiofilm activity against *E. coli* and *P. aeruginosa*	[95]
*Massilia* sp. MAHUQ-52	Antimicrobial	Particle size between 15 and 55 nm Spherical morphology Zeta potential of −18.4 mV SPR peak at 435 nm	Antibacterial activity against drug resistant *Klebsiella pneumoniae* and *Salmonella enteritidis*	[96]
*Streptomyces rochei* SSCM102	Antimicrobial	Particle size between 11 and 21 nm Cubic morphology SPR peak at 380 nm	Antibacterial activity against multiple strains of human pathogens	[97]
*Arthrospira platensis*	Antimicrobial	Average particle size of 50 nm Spherical morphology SPR peak at 450 nm	Antifungal activity against *Aspergillus fumigatus* and *F. oxysporum*	[98]
*Bacillus zanthoxyli* GBE11	Antimicrobial	Particle size between 3.68 and 31.60 nm Spherical morphology Zeta potential of −23.53 ± 1.46 mV SPR peak at 439 nm	Antibacterial activity against multiple strains	[99]
Native bacterium GFCr-4	Environmental Biomedical	Average particle size of 25 nm Spherical morphology SPR peak at 420 nm	Catalytic activity on the production of 2-aminothiophene derivatives Anticancer activity against MCF-7 cancer cells	[100]
*Streptomycetes parvulus* strain K2	Environmental	Particle size between 5 and 45 nm Nearly spherical morphology SPR peak at 420 nm	Water treatment capability on drinking water infected with multiple bacterial strains	[101]
*Bacillus anthracis* PFAB2	Antimicrobial	Average particle size of 84 nm Nearly spherical morphology Zeta potential of −15.5 mV SPR peaks at a range of 350–400 nm	Antibacterial activity against multiple strains Antifungal activity against multiple strains	[102]

**Table 4:** Applications and Properties of Fungi-Based AgNPs.

Synthesized Fungal Strain	Application Type	NP Property	Results	References
*Talaromyces funiculosus*	Antimicrobial Biomedical	Average particle size of 34.32 nm Spherical morphology Zeta potential of −18.41 mV SPR peak at 422.5 nm	Antibacterial activity against multiple strains Antifungal activity against multiple strains Anticancer activity against Hep-G2 and HEK-293 cancer cell lines Antioxidant activity by increasing GSH and reducing MDA levels Anti-inflammatory activity by increasing IL-10 production and reducing TNF-α levels	[103]
*Streptomyces chiangmaiensis* SSUT88A	Antimicrobial	Average particle size of 13.57 nm for Intracellular cell-free supernatant (IS-AgNPs) and 30.47 nm for Extracellular cell-free supernatant (ES-AgNPs) Spherical morphology for both IS- and ES-AgNPs Zeta potential of −32.0 mV for IS-AgNPs and −27.9 mV for ES-AgNPs SPR peaks at 418 nm for IS-AgNPs and 422 nm for ES-AgNPs	Antibacterial activity against multiple drug-resistant strains by IS-AgNPs	[104]
*Nigrospora oryzae*	Antimicrobial	Particle size between 3 and 13 nm Spherical morphology SPR peak at 420 nm	Antifungal activity against *Fusarium* spp.	[105]
*Fusarium* sp.	Antimicrobial	Average particle size of 38.5 nm Spherical morphology SPR peak at 418 nm	Antibacterial activity against multiple strains	[106]
*Penicillium verrucosum*	Antimicrobial	Particle size between 10 and 12 nm Spherical morphology SPR peak at 420 nm	Antifungal activity against *Fusarium chlamydosporum* and *Aspergillus flavus*	[107]
*F. oxysporum* *Fusarium proliferatum*	Antimicrobial Biomedical Agricultural	Particle sizes between 14 to 27 nm for *F. oxysporum* derived AgNPs (FoAgNPs) and 18 to 40 nm for *F. proliferatum* derived AgNPs (FpAgNPs) Spherical to globose morphology for FpAgNPs and rectangular to spherical morphology for FoAgNPs SPR peaks at 450 nm for FpAgNPs and 435 nm for FoAgNPs	Antibacterial activity against multiple strains of human pathogens Antifungal activity against multiple strains Antioxidant activity Larvicidal activity Phyto-stimulatory activity on *Vigna radiata* seeds	[108]
*Aristolochia indica*	Antimicrobial Biomedical	Particle size between 15 and 40 nm Spherical morphology Zeta potential of −70.0 mV SPR peak at 426 nm	Antibacterial activity Antioxidant activity Anticancer activity against MCF-7 cancer cells	[109]
*Lepista sordida*	Antimicrobial	Particle size between 65 to 75 nm Hexagonal morphology SPR peak at 345 nm	Antifungal activity against *Aspergillus flavus* and *Alternaria alternata*	
*Agaricus avensis*	Environmental	Average particle size of 88.49 ± 3.83 nm Zeta potential of −9.16 mV Spherical and irregular morphology SPR peak at 260 nm	Catalytic activity on the conversion of *L*-tyrosine to *L*-dopa	[110,111]
*Thermomyces lanuginosus* BJMDU1	Antimicrobial Environmental	Average particle size of 80 nm Spherical and oval morphology SPR peak at 430 nm	Antibacterial activity against multiple strains Anti-malarial activity Catalytic activity on the conversion of *p*-nitrophenol to *p*-aminophenol	[112]

## Data Availability

No new data were created or analyzed in this study.
